# High diversity and rapid diversification in the head louse, *Pediculus humanus* (Pediculidae: Phthiraptera)

**DOI:** 10.1038/srep14188

**Published:** 2015-09-16

**Authors:** Muhammad Ashfaq, Sean Prosser, Saima Nasir, Mariyam Masood, Sujeevan Ratnasingham, Paul D. N. Hebert

**Affiliations:** 1 Biodiversity Institute of Ontario, University of Guelph, Guelph, ON, Canada; 2 Pakistan Council for Science and Technology, Islamabad, Pakistan; 3 National Institute for Biotechnology and Genetic Engineering, Jhang Road, Faisalabad, Pakistan

## Abstract

The study analyzes sequence variation of two mitochondrial genes (COI, cytb) in *Pediculus humanus* from three countries (Egypt, Pakistan, South Africa) that have received little prior attention, and integrates these results with prior data. Analysis indicates a maximum K2P distance of 10.3% among 960 COI sequences and 13.8% among 479 cytb sequences. Three analytical methods (BIN, PTP, ABGD) reveal five concordant OTUs for COI and cytb. Neighbor-Joining analysis of the COI sequences confirm five clusters; three corresponding to previously recognized mitochondrial clades A, B, C and two new clades, “D” and “E”, showing 2.3% and 2.8% divergence from their nearest neighbors (NN). Cytb data corroborate five clusters showing that clades “D” and “E” are both 4.6% divergent from their respective NN clades. Phylogenetic analysis supports the monophyly of all clusters recovered by NJ analysis. Divergence time estimates suggest that the earliest split of *P. humanus* clades occured slightly more than one million years ago (MYa) and the latest about 0.3 MYa. Sequence divergences in COI and cytb among the five clades of *P. humanus* are 10X those in their human host, a difference that likely reflects both rate acceleration and the acquisition of lice clades from several archaic hominid lineages.

The genus *Pediculus* includes two species—*Pediculus humanus* which occurs only on *Homo sapiens* while *Pediculus schaeffi* occurs on both the bonobo, *Pan paniscus*, and the chimpanzee, *Pan troglodytes*^1^. *P. humanus* includes two morphologically indistinguishable subspecies—the head louse, *P. humanus capitis* De Geer and the body louse, *P. humanus humanus* L.^2^,^3^,^4^. The recovery of lice from mummies^5^,^6^ and evidence of pediculosis in Neolithic remains^7^,^8^ indicate that the relationship between lice and humans has been longstanding^9^. Aside from their role as pests^10^,^11^,^12^, lice are disease vectors^13^,^14^,^15^, justifying a detailed understanding of their diversity.

Both mitochondrial and nuclear genes have been employed to identify lineages and to analyze patterns of genetic diversity in *P. humanus*^5^,^16^,^17^,^18^. Ascunce *et al.*^19^ found regional genetic clustering and inbreeding through the study of sequence variation at 15 nuclear microsatellite loci. Leo & Barker^20^ used small subunit (ssu) rRNA to examine the phylogenetic relationships of head and body lice, while Kittler *et al.*^21^ investigated molecular evolution in *P. humanus* with both nuclear (EF-1α, RPII) and mitochondrial (ND4, cytb) genes. Likewise, Light *et al.*^22^ investigated the taxonomic status of head and body lice using a combination of mitochondrial (COI, cytb, ND4) and nuclear (18S rRNA, EF-1a) loci. Despite this work, the status of head and body lice remains controversial. Most genetic studies have concluded that head and body lice are ecomorphs of the same species^18^,^22^ with body lice originating from head lice^23^. However, other studies have suggested their reproductive isolation^24^, a conclusion supported by the fact that head and body lice possess diagnostic sequence differences in the Phum_PHUM540560 gene^25^.

Aside from providing insights concerning the relationships between head and body lice, prior work has revealed that *P. humanus* includes three genetically distinct lineages. Current understanding of clade diversity and distribution is largely based on studies of sequence variation in two mitochondrial genes, COI and cytb. These studies have shown that clade A possesses a global distribution, while clade B occurs in Europe, Australia and the New World, and clade C is only known from Africa^26^,^27^. These distributional patterns are based on reasonably detailed work in Europe and the New World^4^,^9^, but patterns of genetic diversity in Asian and African head lice have seen much less study. Prior work has suggested that the known lice clades evolved on different hosts^9^, likely the several lineages of *Homo* known from 2.3 to 0.03 million years ago (MYa)^28^. Considering the diversity of prehistoric hominids and the limited phylogeographic information on human lice, expanded sampling is imperative to fully understand the historical relationship between humans and lice. The current study begins to address this gap by examining sequence diversity in COI and cytb for a large number of lice from Pakistan and through smaller-scale investigations in Egypt and South Africa. These results are integrated with prior data to expand perspectives on the number, distributions, and diversification rates of clades of *P. humanus*.

## Results

### Sequence analysis

**Figure 1:**
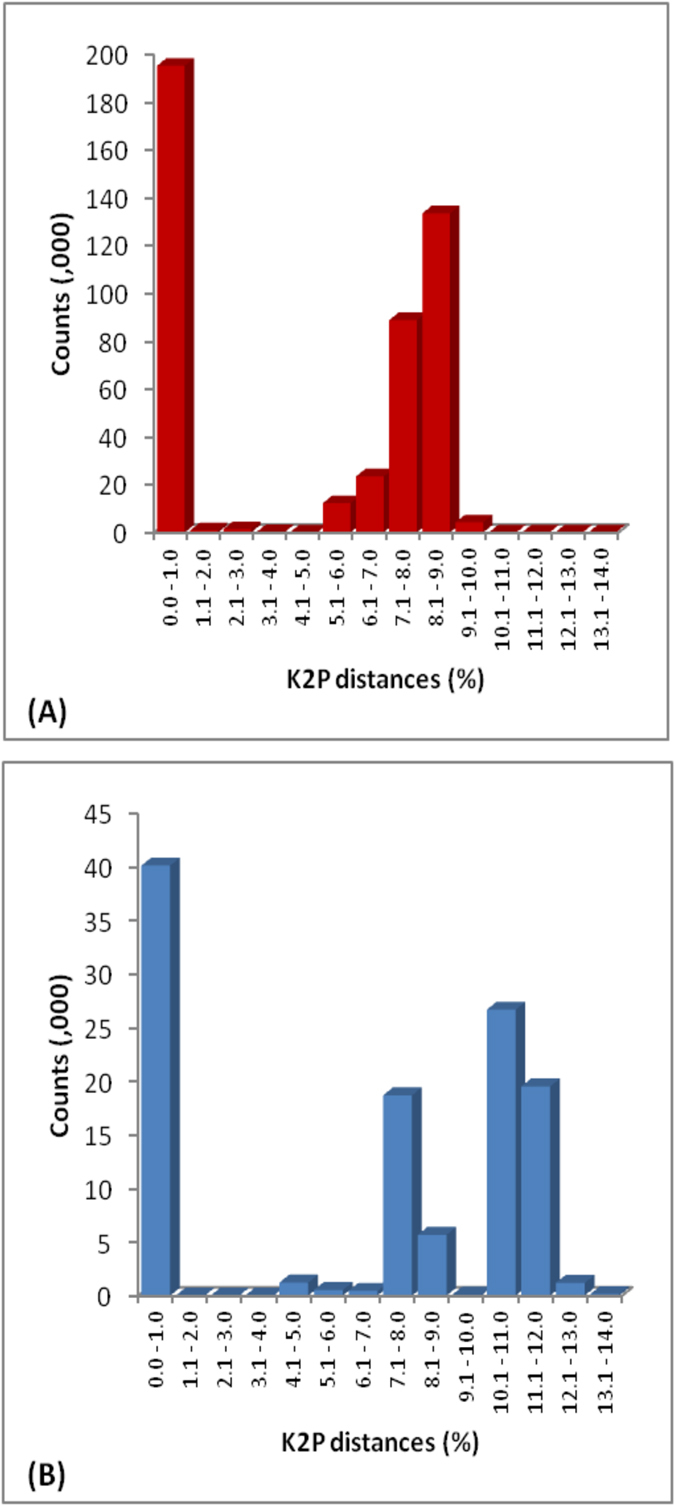
Histograms of pairwise (K2P) distances among 960 COI (A) and 479 cytb (B) sequences from head lice.

Barcode (COI-5′) sequences (>500 bp) were recovered from 693 of 842 lice and were then aligned with 267 COI accessions from GenBank to create a dataset of 960 sequences which was used for subsequent analysis. The resulting alignment showed an overlap of 453–658 bp between COI sequences from this study and those from GenBank. Our collections provided 563 COI records from Pakistan, 81 from Egypt, 45 from South Africa, 3 from Honduras and 1 from Canada while the 267 GenBank accessions included specimens from 30 countries and a few from unknown locations (Table S2). Our cytb dataset included 239 sequences (89 from Pakistan, 89 from Egypt, 57 from South Africa, 4 from Honduras) which were aligned with 240 cytb accessions from GenBank (22 countries) creating a dataset of 479 sequences. Except for 28 records with 272 bp of overlap, all cytb sequences from GenBank possessed 294 bp of overlap with the sequences obtained in this study. Kimura-2-parameter (K2P) distances among the COI and cytb sequences ranged from 0.0–10.3% (mean: 4.6% ± 0.6) and 0.0–13.8% (mean: 6.6% ± 1.1), respectively. Most pairwise barcode distances fell between 0.0–1.0% or 5.0–10% (Fig. 1A) with none between 3.8–4.5%. Similarly, most cytb distances were distributed within 0.0–1.0% and 7.0–12% (Fig. 1B) with none between 1.4–4.6%.

The “Basic Local Alignment Search Tool (BLAST)” on GenBank and the “Identification Request” on Barcode of Life Data Systems (BOLD)^29^ were used to identify matches to the sequences generated in this study. The matching GenBank accessions were traced to the relevant publications and the matching BOLD process IDs were traced to BINs (Barcode Index Numbers)^30^ to confirm the BIN assignment for each clade. BLAST analysis of the new COI sequences revealed matches (99%–100% nucleotide identity) to two lice clades (A, B) with all 81 sequences from Egypt, 25 from Pakistan, and the sole sequence from Canada matching clade A while the 3 sequences from Honduras and 45 from South Africa matched clade B. The barcode “Identification Request” on BOLD showed that the sequences matching clade A were assigned to BIN:AAA1556 while those matching clade B were in BIN:AAA1557. The other 538 sequences from Pakistan which lacked a close GenBank match were assigned to BIN:AAW5034. As our barcode data revealed three divergent lineages at COI, we sequenced cytb from selected specimens of each lineage, performed a “BLAST Search” and harvested the corresponding sequences from GenBank. Based on their barcode association, the cytb sequences were assigned to the known lice clades.

### Lice clades

The global COI and cytb datasets were analyzed using the Barcode Index Number (BIN) system^30^, Poisson Tree Processes (PTP)^31^, and Automatic Barcode Gap Discovery (ABGD)^32^ to ascertain the number of OTUs (Table 1). The BIN system assigned the 960 COI sequences to five BINs (AAA1556, AAA1557, AAA1558, AAW5034, ACR6059). The first three BINs corresponded to the three known head lice clades (A, B, C) while the fourth (AAW5034) and fifth (ACR6059) were new additions. A standalone version of RESL, the algorithm that underpins BIN assignments, was also used to ascertain the number of OTUs in the cytb data. This approach assigned the 479 cytb sequences to five OTUs (Table 1) with three corresponding to the known clades (A, B, C) while the fourth corresponded to the new clade “D”, and the fifth to a clade “E” which was represented by one sequence from Ethiopia (AY316774)^21^ and four (KC685774, KC685782, KC685784, KC685786) from unknown locations. PTP assigned the COI and cytb datasets to five OTUs in both the maximum likelihood (ML) and Bayesian partitions (Suppl. 1) which were congruent with the OTUs recovered by the BIN algorithm (Table 1). The ABGD determines the initial and recursive partitions in the sequences for varied prior maximal distances (PMD). Analysis of COI sequences showed four initial and five recursive partitions with PMDs ranging from 0.0028–0.0129, three initial and five recursive at 0.0215, but just three initial and recursive partitions with PMD 0.0359 (Table 1). The ABGD analysis for cytb showed five initial and six recursive partitions with a PMD of 0.0046 and five initial and recursive partitions with PMDs ranging from 0.0129–0.0359 (Table 1).

### Phylogenetic analysis and divergence time estimates

**Figure 2:**
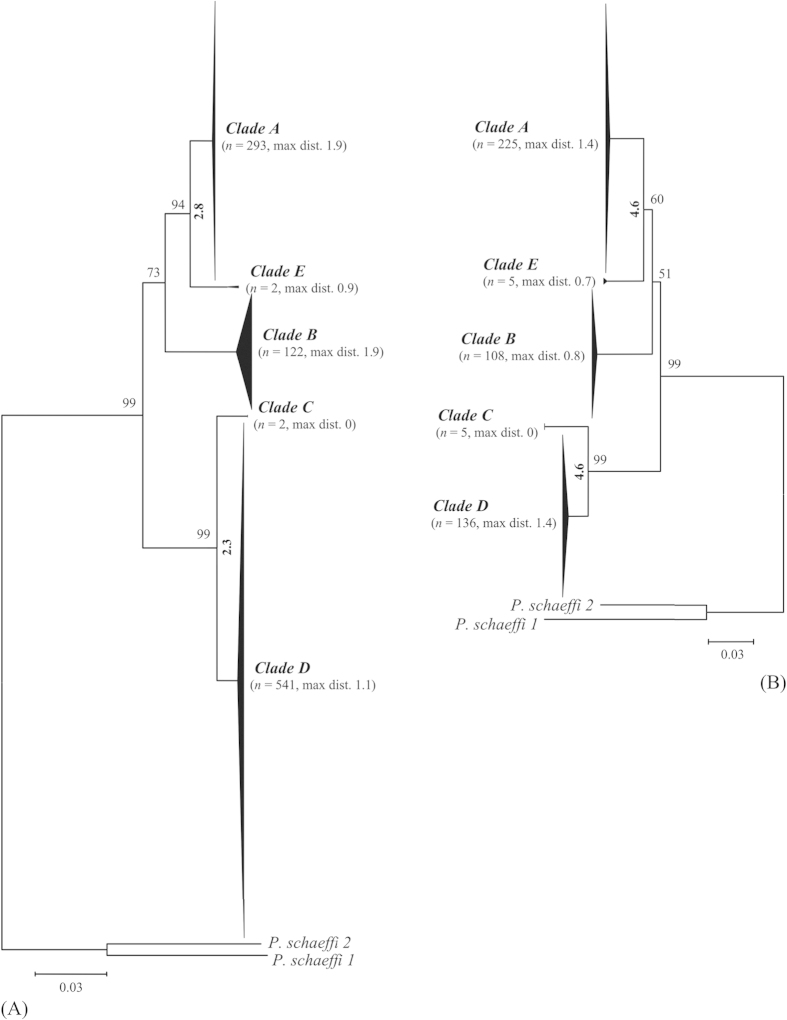
NJ cluster analysis of 960 COI (A) and 479 cytb (B) sequences from head lice. Bootstrap values (500 replicates) are shown above the branches. The scale bar shows K2P distances. The node for each clade with multiple specimens is collapsed to a vertical triangle, with the horizontal depth indicating the level of intraclade divergence. Bracketed numbers next to each clade name indicate the number of individuals analyzed and the maximum intraclade distance. Analyses were conducted in MEGA5.

**Figure 3:**
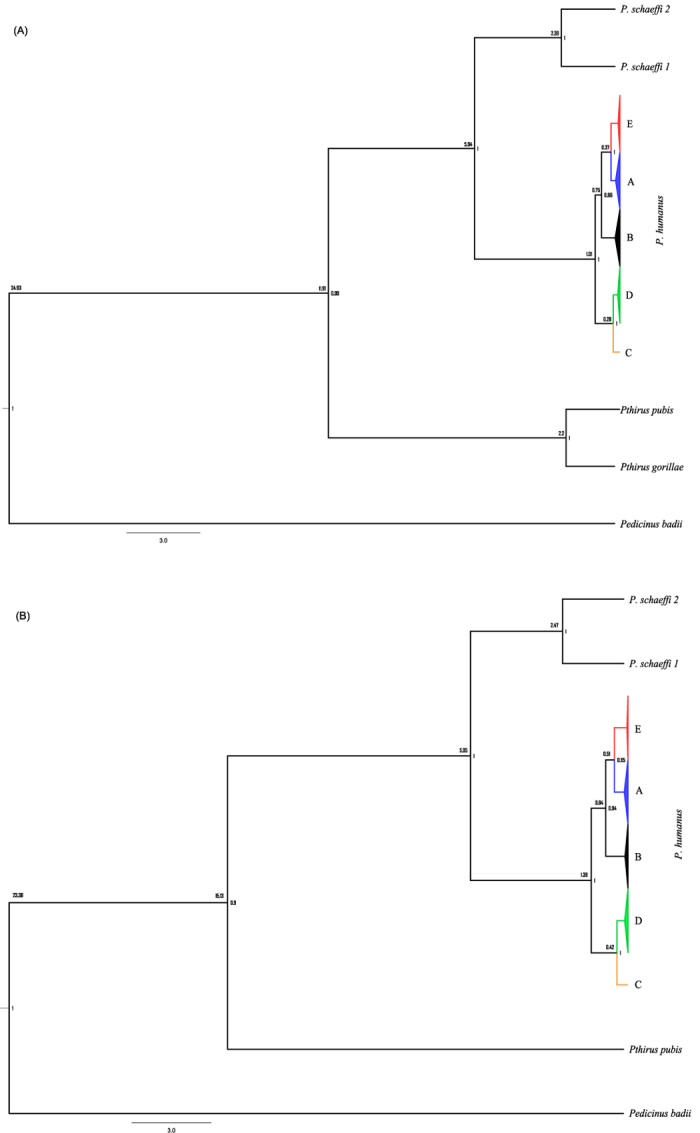
Phylogenetic and divergence time analysis of COI (A) and cytb (B) sequences for head lice clades, including lice from other primates, using Bayesian inference and BEAST. Posterior probabilities are indicated inside while divergence time (million years) outside the nodes. The node for each clade with multiple sequences is collapsed.

NJ analysis of COI (Fig. 2A) and cytb (Fig. 2B) showed that the sequences in each clade formed a monophyletic cluster with maximum intracluster distances at COI of 1.9%, 1.9% and 1.1% for clades A (*n* = 293), B (*n* = 122) and “D” (*n* = 541), respectively. The two sequences for “E” showed 0.9% divergence, while those for clade C lacked variation. The maximum intracluster distances at cytb were 1.4%, 0.8%, 0.0%, 1.4% and 0.7% for clades A (*n* = 225), B (*n* = 108), C (*n* = 5), “D” (*n* = 136) and “E” (*n* = 5) respectively. The NN distances between clades and the nodal supports are presented in Fig. 2A (COI) and 2B (cytb). The phylogenetic trees estimated by Bayesian evolutionary analysis (Fig. 3A,B) and maximum parsimony (MP) (Fig. S1) possessed similar node patterns to those recovered with NJ analysis. The posterior probabilities for all nodes in the COI (Fig. 3A) and cytb (Fig. 3B) trees from Bayesian evolutionary analysis were greater than 85%. The MP-inferred bootstrap support (Fig. S1) for clade “D” was 99% at both COI and cytb, while for clade “E” it was 88% at COI and <50% at cytb. The posterior probabilities from Bayesian inference determined with another substitution model, GTR + G, were 100% for clade “D” and over 50% for clade “E” (Fig. S2) at both COI and cytb. The divergence time estimates for *P. humanus* clades are presented in Fig. 3A (COI) and 3B (cytb). The estimates were made by calibrating divergence time with *P. humanus - P. schaeffi* split along with their respective primate hosts. The estimates showed that the first split of *P. humanus* clades occurred over 1.0–1.4 MYa and the last less than 0.3–0.4 MYa. The divergence time estimate for the most recent common ancestor (MRCA) of *P. schaeffi 1 - P. schaeffi 2* was 2.4 MYa for COI and 2.5 MYa for cytb while that for the genus *Pediculus* and *Pthirus* was 12 Mya for COI and 15 Mya for cytb.

### Genetic diversity and haplotype analysis

**Figure 4:**
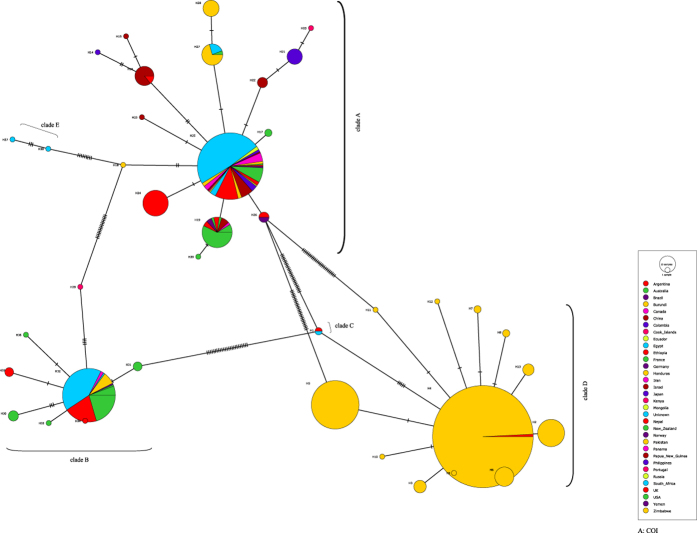
COI haplotype networks of head lice. Each circle indicates a unique haplotype and variation in circle size reflects the number of sequences assigned to a haplotypes. Pie colors and sizes in circles represent the countries and the number of their sequence records for a haplotype.

**Figure 5:**
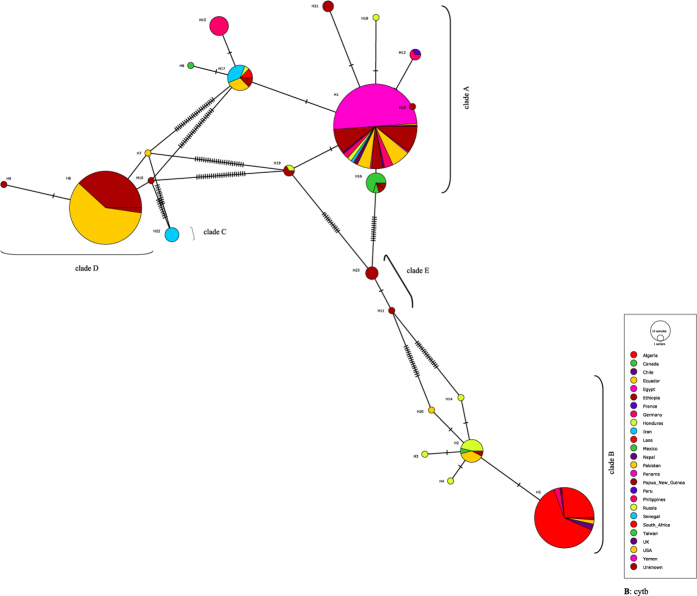
Cytb haplotype networks of head lice. Each circle indicates a unique haplotype and variation in circle size reflects the number of sequences assigned to a haplotypes. Pie colors and sizes in circles represent the countries and the number of their sequence records for a haplotype.

Genetic diversity indices and the results of neutrality tests for COI and cytb are shown in Table 2. The average number of pairwise nucleotide differences (*k*), nucleotide diversity (π) and haplotype diversity (Hd) varied among the clades of both genes. Overall, both (*k*) and (π) were similar in COI and cytb (Table 2). AMOVA revealed significant variation among clades and among populations with high F_st_ values for both COI and cytb (Table 3). The COI and cytb sequences were analyzed to determine the number of haplotypes in the dataset and in each clade and their distributions (Figs 4 and 5). 39 COI haplotypes were detected including 16 in clade A (from 28 countries and 1 unknown location) with the dominant haplotype (57%) found in 21 countries. Clade B included 8 COI haplotypes in 10 countries with the dominant one (89%) present in nine countries. Clade “D” included 12 COI haplotypes with the dominant haplotype (72%) shared by Pakistan (*n* = 389) and Nepal (*n* = 3) (Fig. 4). Cytb sequences revealed 23 haplotypes from 25 countries with 10 for clade A (23 countries), 6 for clade B (9 countries), 4 for clade D (2 countries) and 2 for clade E (one country) (Fig. 5). One cytb haplotype for clade A was dominant (79%), being detected in 18 countries while the commonest haplotype (80%) of clade B was found in six countries. Among four haplotypes of clade “D”, one was dominant (98%), being found in Ethiopia and Pakistan (Fig. 5).

### Sequence divergence in Pediculus and its hosts

**Figure 6:**
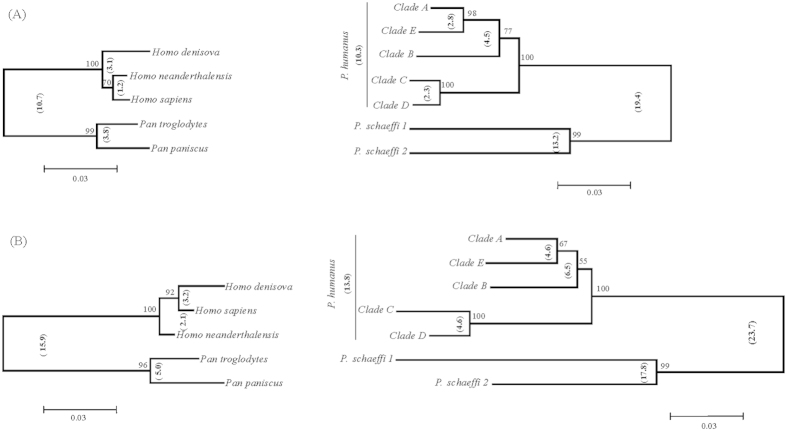
COI (A) and cytb (B) NJ analysis of hominids (left) and their parasitic lice (right). Numbers in brackets between tree branches indicate nearest-neighbor K2P distances.

Levels of sequence divergence at both COI and cytb between the two species of *Pediculus* were much higher than those between their hosts (Fig. 6). The two recognized species of *Pediculus* show a minimum divergence of 19.4% (COI) and 23.7% (cytb) while chimpanzees/bonobos and humans show 10.7%/11.5% (COI) and 15.9%/16.8% (cytb) divergence (Fig. 6). Interestingly, *P. schaeffi* included two lineages with 13.2% divergence at COI and 17.8% at cytb while its hosts (chimpanzee, bonobo) show only 3.8% divergence at COI and 5% at cytb (Fig. 6). Accessions indicate that *P. schaeffi 1* (AY695999, AY696067) was collected from *P. troglodytes*, but host information for *P. schaeffi 2* (KC241887, KC241883) is lacking, although it might well occur on *P. paniscus*. The evidence for differential levels of sequence divergence between hosts and parasites was particularly striking for *P. humanus* as its maximum divergence was 10.3% at COI, versus just 0.9% for *H. sapiens* or 3.1% with the inclusion of sequences for *Homo s. neanderthalensis* and *Homo s. denisova*. The same pattern was evident for cytb with 13.8% divergence for *P. humanus* versus 1% in *H. sapiens* or 3.2% with the inclusion of *H. s. neanderthalensis* and *H. s. denisova.*

The evidence for much more rapid diversification in lice than their hosts is further reinforced by comparison of AA sequences. The lineages of *Pediculus* possess numerous AA substitutions in cytb and six in COI (Fig. S3), while species of *Homo* and *Pan* possess only 10 AA substitutions for cytb and one for COI.

## Discussion

The genus *Pediculus* is currently recognized as including just two species, *P. schaeffi* and *P. humanus*. The MRCA for these two lice species likely existed about 6 MYa, the estimated date of divergence of their hosts^33^. These lice show a minimum 19.4% divergence at COI and 23.7% at cytb, values twice as high as those between their human and chimpanzee hosts, indicating the rate acceleration noted between other lice species and their hosts. For example, mtDNA genes in lice parasitic on birds evolve about 3X as rapidly as those in their hosts^34^. A recent comparative analysis^35^ of 1534 protein-coding genes from humans, chimpanzees and their two species of *Pediculus* revealed that rates of DNA substitution in nuclear genes were, on average, 14X faster in lice than in their hosts, while mitochondrial genes showed just 2.9X acceleration. While Johnson *et al.*^35^ reached conclusions based on interspecific comparisons, the present study extends the evidence for rate acceleration by revealing the much higher levels of intra-specific sequence divergence in *P. humanus* than in *H. sapiens*. The five clades of *P. humanus* have maximum divergences of 10.3% at COI and 13.8% at cytb, 10-fold higher than those in human populations (0.9% at COI, 1% at cytb).

The present study has revealed the need for more detailed study of *P. schaeffi* to ascertain if its two deeply divergent lineages are sibling taxa, one restricted to *P. troglodytes* and the other to *P. paniscus.* Because the three subspecies of *P. troglodytes* have little gene flow^36^, it would also be interesting to examine the possibility that each hosts a distinct clade of lice as this information would aid interpretation of the sequence variation seen among lineages in *P. humanus*. Because their hosts diverged about 2 MYa^37^, studies of sequence divergence among the lice lineages on chimps and bonobos provide a good system for helping to understand the origins of the five clades in *P humanus*. Firstly, they confirm that rates of mtDNA evolution have been about three times higher in lice than in their primate hosts, as the two lineages of *P. schaeffi* show 13.2% divergence at COI and 17.8% at cytb, versus the 3.8% and 5.0% divergences between chimps and bonobo. Secondly, the close congruence in divergence between lineages of *P. schaeffi* and the maximum divergences (10.3% at COI, 13.8% at cytb) in *P. humanus* suggests similar histories of diversification. As the split between chimpanzees and bonobos occurred approximately 2 MYa, lineages of *P. humanus* are likely to have diverged more than 1MYa.

This study investigated levels of sequence divergence in two mitochondrial genes in *P. humanus* from regions of Africa and Asia which have seen little prior investigation. By coupling these results with other data, the present study has expanded understanding of its levels and patterning of sequence divergence. Three analytical methods (BINs, PTP, ABGD) support the presence of five OTUs at both COI and cytb, results congruent with the phylogenetic analysis. Although three mitochondrial clades of head lice have generally been accepted^26^, Ascunce *et al.*^19^ reported four geographically structured clusters based on the analysis of variation in nuclear microsatellites. The present study provides additional evidence for high diversity in head lice, suggesting the occurrence of five mitochondrial lineages. A prior study on head lice^22^ found maximum distances within clades of 1.0% (COI) and 0.4% (cytb), while NN distances at these genes were 5.9% and 6%, respectively. In this study maximum distances within clades were higher (COI: 1.9%; cytb: 1.4%) while NN distances were lower (COI: 2.3%; cytb: 4.6%), reflecting the larger sample sizes and extended geographic coverage that led to the recovery of additional haplotypes within each clade.

Previous studies have shown that clade A has a global distribution^5^,^26^, results supported by our detection of it in Canada, Egypt and northern Pakistan. Past work has indicated that clade B is limited to Australia, Europe and North America with evidence suggesting that it colonized Europe and Australia from North America^26^. Light *et al.*^26^ suggested the likelihood of further expansion in its range, perhaps explaining our detection of clade B in South Africa, and by others in Algeria (Table S2). Our sampling did not encounter any new specimens of clade C so it remains restricted to Africa, but our results establish that clade “D” is the sole or dominant lineage in most regions of Pakistan and other records indicate its occurrence in Nepal and Ethiopia. The final clade, “E”, is known from two specimens for COI and five for cytb, but only one has locality information—Ethiopia. More detailed analysis of sequence diversity in *P. humanus* is required to clarify the origins and current distributions of its major lineages.

Although clade A was also detected in Pakistan, clade “D” was dominant lineage in this region. The earliest record for *Homo* in Pakistan is dated to 1.9 MYa with a gap until around 45,000 years before present (YBP)^38^,^39^. The divergence of clade C from the MRCA of clades A and B was estimated as occurring just over 1.0 MYa while that of clade “D” from its MRCA occurred around 0.4–0.3 MYa. Interestingly, DNA studies^40^ have placed the divergence time between the MRCA of *H. sapiens/H. s. neanderthalensis* and *H. s. denisova* at about 1.0 MYa and that between *H. sapiens* and *H. s. neanderthalensis* at about 0.47 MYa. Our time estimates on the split between *Pedicinus badii* and the MRCA of *Pthirus* and *Pediculus* (24.9 MY (COI)–23.5 MY (cytb)), and that between *Pthirus* and *Pediculus* (12 MY (COI)–15 MY (cytb)) are very close to those reported in other studies (22.5 MY and 13 MY)^9^,^33^. Our estimated divergence date for the MRCA of the two lineages of *P. schaeffi* (*P. schaeffi 1, P. schaeffi 2*) was 2.4 MYa, reflecting the date estimated for the split between chimps and bonobos^37^. This validates our analytical approach and supports the conclusion on divergence time estimates for *P. humanus* clades. Combined records, from GenBank and this study, show that all the lice clades, including “D” and “E” are found in Africa, but some of these occurrences (e.g. clade B) may represent recent range expansions linked to the rise in travel.

**Figure 7:**
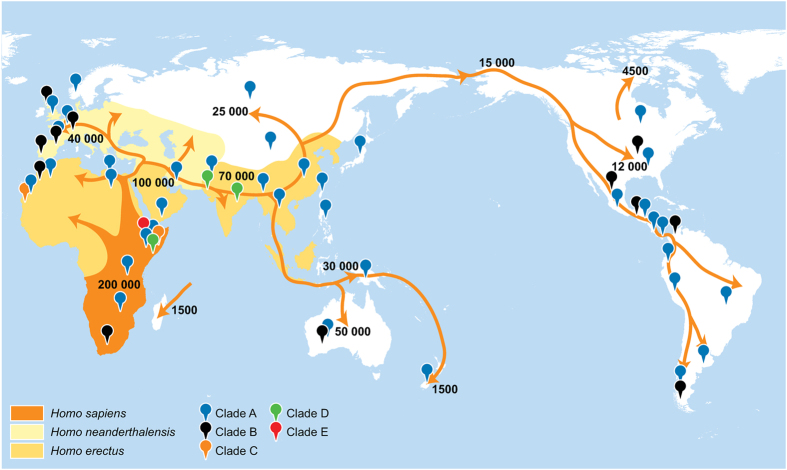
A map of early human migration patterns and the distribution of *Pediculus humanus* clades.

Molecular analyses of archaic hominids suggest at least three waves of dispersal, the first about 1.0 MYa which led to establishment of the Denisovans. A second dispersal event about 0.6 Mya led to the establishment of the Neanderthals, while the third involved modern humans. The earliest records for modern *H. sapiens* appear in the fossil record in Ethiopia around 200,000 YBP, but our species only colonized Eurasia about 70,000 and Australia 50,000 YBP^41^. The high levels of sequence divergence among lice lineages on modern humans strongly suggests the ‘inheritance’ of lineages that evolved in association with archaic hominids but subsequently shifted hosts. The distributional patterns of lice clades and of human dispersal (Fig. 7) indicate that *H. sapiens* might have co-existed with *H. erectus* and *H. s. denisova* in Asia and with *H. s. neanderthalensis* in Europe, creating a scenario that could have led to the acquisition of lice lineages from each of these hosts, an hypothesis discussed in other reports^42^. However, our detection of additional clades of *P.humanus* suggests their acquisition from other archaic lineages, such as Denisovan man^40^.

The low mitochondrial sequence variation in human populations^43^ has been linked to a severe population bottleneck about 70,000 years ago when human populations may have declined to a few thousand individuals^44^. If a single clade of *P. humanus* survived this collapse, it would likely (based upon current levels of intra-clade variation) have possessed a maximum sequence divergence of about 1.5%. The fact that *P. humanus* includes five clades with up to 13.8% divergence at cytb would require sequence divergence 100X faster than usual rates of mitochondrial change (2% per million years) if all modern lineages derive from a single clade that survived this bottleneck, far greater than the 3X acceleration detected in other studies of rate acceleration in lice. Alternatively, each of the five lice clades may have evolved on another hominid lineage, ‘jumping ship’ as its host declined. Reed *et al.*^9^ proposed this explanation for the two lineages of *P. humanus* recognized at that time, suggesting that one evolved on *H. sapiens*, while the other was originally parasitic on *Homo erectus*. The larger number of lice lineages revealed by this study complicates this explanation, but it is possible that modern humans acquired lice from diverse archaic hominid lineages, including Neanderthal and Denisovan man as well as *H. erectus*. Evidence for gene flow between archaic hominids and *H. sapiens* is strong^45^,^46^, ensuring that the close body contact needed to facilitate parasite transfers was met. There are, as well, reports^47^ of humans acquiring divergent lineages of parasites from other primates. For example, humans are attacked by two types of *Plasmodium knowlesi*, one from *Macaca fascicularis* and the other from *M. nemestrina*. More detailed study of the geographic variation in lice lineages, especially those associated with ancient human remains, could help to clarify the possibility that *H. sapiens* not only gained new genes, but also new parasites as it displaced other hominids.

## Methods

### Specimen collections

837 head lice were analyzed including 686 specimens from 277 individuals in Pakistan, 91 specimens from three individuals in Egypt, and 60 specimens from an unrecorded number of individuals in South Africa. All specimens were preserved in 95% ethanol before being assigned a specimen number and photographed. Specimen data along with collection information are available in the project MAPED (*Pediculus humanus* mitochondrial diversity) on BOLD. All specimens from Egypt and South Africa as well as 89 specimens from Pakistan were also analyzed for cytb. In addition, COI-5′ and cytb sequences (267 and 240, respectively) of *P. humanus* (collected in 37 countries) were extracted from GenBank to gain a better understanding of the geographical patterning of genetic diversity in head lice.

### DNA isolation, PCR amplification and sequencing

Each specimen was transferred to a well pre-loaded with 30 μl of 95% ethanol in a 96-well microplate and DNA was extracted at the Canadian Centre for DNA Barcoding (CCDB) using standard protocols^48^. PCR amplification and sequencing were subsequently performed at the CCDB, also following standard protocols^49^. Amplification of the COI-5′ (barcode) employed the primer pair LCO1490_t1 (TGTAAAACGACGGCCAGTGGTCAACAAATCATAAAGATATTGG) and HCO2198_t1 (CAGGAAACAGCTATGACTAAACTTCAGGGTGACCAAAAAATCA) using the following PCR conditions: 94 °C (1 min); 5 cycles of 94 °C (30 s), 45 °C (40 s), 72 °C (1 min); 35 cycles of 94 °C (30 s), 51 °C (40 s), 72 °C (1 min); and final extension of 72 °C (10 min). Amplification of cytb was performed with primer pair Ph-cytb-F2 (GGCGACTGTTATTACTAACCT) and Ph-cytb-R2 (TCCGGGTCCATGAAGAAGTCC) using the same PCR conditions. PCRs were carried out in 12.5 μL reactions with the standard CCDB reaction cocktail and 2 μL of DNA template. PCR products were visualized on a 2% agarose E-gel^®^ 96 system (Invitrogen Inc.) and amplicons were bidirectionally sequenced using BigDye Terminator Cycle Sequencing Kit (v3.1) on an ABI 3730XL DNA Analyzer. The forward and the reverse sequences were assembled, edited and aligned using CodonCode Aligner (CodonCode Corporation, USA) and translated in MEGA (V5)^50^ to verify that they were free of stop codons and gaps. All sequences generated in this study and their GenBank accession numbers (Table S2) are accessible on BOLD in the dataset DS-MAPTH and in dx.doi.org/10.5883/DS-MAPTH.

### Sequence analysis

COI and cytb sequences obtained in this study were combined with those for head lice from GenBank to generate a global dataset to examine clade diversity in *P. humanus*. Because GenBank sequences for the two gene regions from individual lice could not be linked, the results for each gene were analyzed separately. ClustalW alignments were performed in MEGA5 and the non-overlapping sequence regions at the 5′- and 3′-ends were trimmed. Alignments were exported as fasta files and K2P pairwise distances were calculated using MEGA5 with pairwise deletion of gaps and missing data.

### Delimitation of lice clades

Molecular operational taxonomic units (OTUs) have frequently been used to infer putative species boundaries where morphological identifications are difficult^51^,^52^. We used three approaches to assign the COI and cytb sequences from head lice to OTUs: BIN system^30^, PTP^31^, and ABGD^32^. The BIN system uses the RESL algorithm to reach decisions on the number of OTUs in a sequence dataset through a three-phased analysis^30^. It starts with single linkage clustering using a fixed 2.2% threshold, followed by Markov clustering, which aims to improve the accuracy of the OTUs, and finally employs the Silhouette criterion to compare the different clustering schemes from Markov clustering, selecting the option with the highest Silhouette score. A stand-alone version of RESL was employed to analyze both the COI and cytb datasets. The PTP delimits OTU boundaries based on rooted phylogenetic trees with speciation and branching events modeled by maximum-likelihood and Bayesian support examining the number of substitutions^31^. This model has been integrated with the evolutionary placement algorithm (EPA-PTP) to estimate the number of OTUs in phylogenetic placements. The PTP analysis was performed first by generating a ML tree in MEGA5 and then exporting the tree as a Newick file, which was subsequently used in an online version of PTP (http://species.h-its.org/ptp/) to infer the number of OTUs. PTP generates two solutions; 1) Maximum Likelihood (ML) (# of max likelihood partitions), and 2) Highest Bayesian (# of most supported partitions found by simple heuristic search). These partitions were assumed equivalent to the number of OTUs in each (COI, cytb) dataset. ABGD employs a multi-phase system which initially divides sequences into OTUs based on a statistically inferred barcode gap (i.e., initial partitioning), and subsequently conducts additional rounds of splitting (i.e., recursive partitioning). ABGD has three key parameters: (i) *X*, which is an estimate of relative gap width, and (ii) minimum and (iii) maximum values of prior intraspecific divergence (*P*). The COI and cytb sequences were analyzed using an online version of ABGD (http://wwwabi.snv.jussieu.fr/public/abgd/abgdweb.html) employing a relative gap (X) of 1.5, a minimal intraspecific distance (Pmin) of 0.001 and a maximal intraspecific distance (Pmax) ranging from 0.02 to 0.1 employing K2P as the distance metric.

### Phylogenetic analysis

Neighbor-joining (NJ) analysis was performed in MEGA5 using the K2P model with pairwise-deletion and 500 bootstrap replicates. All positions containing gaps and missing data were eliminated. The subtree for each clade of lice was collapsed with the “compress/ expand subtree” function. COI and cytb sequences from *P. schaeffi* (AY695999, KC241887 and AY696067, KC241883) were employed as outgroups. Bayesian evolutionary analysis and divergence time estimates were carried out in BEAST (v1.7.5)^53^. The number of distinct haplotypes was first determined for each gene region in DnaSP (v5.10)^54^ and the unique haplotypes were used in subsequent phylogenetic analyses, reducing computational time and producing visually compact trees. COI and cytb sequences from *P. schaeffi* (*P. schaeffi 1, P. schaeffi 2*), *Pthirus gorillae* (EF152555), *Pthirus pubis* (EF152554, FJ267435), and *Pedicinus badii* (EF152556, FJ267436) were included for the phylogenetic and divergence time analysis. The divergence times for lice clades were estimated in BEAST by using the calibration point of 6 million years (6 ± 0.5) for the split between *P. humanus* and *P. schaeffi* as the MRCA of their corresponding hosts lived about 6 MYa^55^. Researchers have used host divergence to calibrate the lice phylogenetic trees as lice are known to cospeciate with their hosts^33^. The HKY + G model was implemented, and the three codon positions were unlinked in order to be estimated independently. The model selection was made using FindModel (www.hiv.lanl.gov/cgi-bin/findmodel/findmodel.cgi). The calibration priors were modeled as normal distributions. BEAST analyses were performed under a lognormal relaxed clock (uncorrelated) assuming a normal distribution. A Birth-Death speciation process model^56^ was selected for the tree prior and the MCMC analysis were run for 10 million generations with the Markov chain sampled every 1000 generations. Log and tree files from two independent runs were combined in LogCombiner (in BEAST package) with initial 10% trees discarded as burn-in. Tracer v1.6 was used to determine convergence, to measure the effective sample size of each parameter, and to calculate the mean and 95% highest posterior density (HPD) intervals for divergence times. The consensus tree was compiled with TreeAnnotator v1.7.5 and displayed in FigTree v1.4.2. Bayesian inference with the GTR + G model was carried out using MrBayes (v3.2.0)^57^ and the MCMC technique. The data were partitioned in two ways: a single partition with parameters estimated across all codon positions, and a codon-partition in which each codon position was allowed different parameter estimates. The analyses were run for 10 million generations using four chains with sampling every 1,000 generations. Bayesian posterior probabilities (PP) were calculated from the sample points once the MCMC algorithm began to converge. Convergence was determined as the point where the standard deviation of split frequencies declined below 0.05 and the PSRF (potential scale reduction factor) approached 1. Both runs converged on a stationary distribution after the burn-in stage (discarded the first 25% of samples). MP analyses were performed in MEGA5 and the most parsimonious trees were selected. The trees were obtained using the Subtree-Pruning-Regrafting (SPR) algorithm^58^ with search level 1 in which the initial trees were obtained by the random addition of sequences (10 replicates). The percentage of replicate trees in which the associated taxa clustered together in the bootstrap test (500 replicates) was determined.

### Genetic diversity and haplotype analysis

ClustalW alignments (fasta files) were imported into DnaSP to perform genetic diversity analysis and neutrality tests (Fu & Li’s D^59^ and Tajima’s D^60^). Haplotype data were subsequently exported and saved as an Arlequin file. Sites with gaps and missing data were not considered. Analysis of Molecular Variance (AMOVA) was performed in Arlequin (v3.5)^61^ treating samples from each country as one population and from each clade as one group. A minimum spanning network^62^ for COI or cytb, based on haplotype data from DnaSP, was constructed in PopART (http://popart.otago.ac.nz) to visualize the network of interrelationships between the haplotypes.

### Comparison of rates of sequence divergence

Sequences for *P. schaeffi* (COI: AY695999, EF152553, KC241887; cytb: AY696067, KC241883, FJ267434; AY316793), *H. sapiens* (HM771214), *H. s. neanderthalensis* (Neanderthal) (KC879692), *H. s. denisova* (FN673705), *P. troglodytes* (chimpanzee) (JF727202) and *P. paniscus* (Bonobo) (JF727220) were obtained from GenBank to allow a comparison of rates of mitochondrial divergence in species of *Pediculus* and their hosts. After initial scrutiny, one of the cytb sequences (AY316793) was excluded from analysis as it showed very high sequence divergence (59%) from other records for *Pediculus* (and closer similarity to sequences from the dipteran family Chironomidae), suggesting that it is a contaminant. Similar gene regions (COI: 658 bp; cytb: 294 bp) from all the accessions were aligned (ClustalW) in MEGA5 and K2P pairwise distances were calculated with pairwise deletion of gaps and missing data. NJ analyses were performed in MEGA5 using the K2P model with pairwise-deletion and 500 bootstrap replicates. Amino acid (AA) translations were performed and aligned in MEGA5 employing the same parameters, exported as fasta files and analyzed in MegAlign (DNASTAR, Inc. USA) for AA substitutions. COI and cytb divergences in *H. sapiens* were determined using sequences in Ingman *et al.*^63^ available at Human Mitochondrial Genome Database (http://www.mtdb.igp.uu.se/) which derive from individuals from all major language groups.

### Data accessibility

All sequences generated in this study are available on BOLD (www.boldsystems.org) in the data set (DS-MAPTH) and also through the following DOI (dx.doi.org/10.5883/DS-MAPTH). All new sequences have also been deposited in GenBank under the following accession numbers: COI (KJ839836 to KJ840549); cytb (KJ840550 to KJ840795). Additional supporting information including collection locations, photographs, sequences and trace files are available on BOLD.

## Additional Information

**Accession codes**: All new sequences have also been deposited in GenBank under the following accession numbers: COI (KJ839836 to KJ840549); cytb (KJ840550 to KJ840795).

**How to cite this article**: Ashfaq, M. *et al.* High diversity and rapid diversification in the head louse, *Pediculus humanus* (Pediculidae: Phthiraptera). *Sci. Rep.* **5**, 14188; doi: 10.1038/srep14188 (2015).

## Supplementary Material

Supplementary Information

## Figures and Tables

**Table 1:** OTUs recovered from global COI and cytb datasets of *Pediculus humanus* by three different species delimitation methods.

Marker	BIN System			**ABGD**											
				PTP Model		0.0017*		**0.0028**		**0.0077**		**0.0129**		**0.0215**		**0.0359**	
		**ML partition**	**Bayesian partition**	**I**	**R**	**I**	**R**	**I**	**R**	**I**	**R**	**I**	**R**	**I**	**R**		
CO1	5	5	5	4	5	4	5	4	5	4	5	3	5	3	3
Cytb	5	5	5	28	28	28	28	5	5	5	5	5	5	5	5

^*^prior intraspecific divergence (*P*).

I: Initial partitions R: Recursive partitions. Number of ABGD partitions obtained by JC and K2P models was the same. Relative gap width (X) was 1.5.

**Table 2:** Genetic diversity indices and neutrality tests (Fu & Li’s *D* and Tajima’s *D*) on the mitochondrial COI-5′ (barcode) and cytb sequences of *Pediculus humanus*.

**Locus, range**	***n***	**S**	**k**	**π**	**h**	**Hd**	**Fu & Li’s *D* (Significance)**	**Tajima’s *D***
COI, all	960	59	15.58	0.047	39	0.779	0.50 (P > 0.1)	2.20
COI, Clade A	293	20	1.26	0.0038	17	0.74	−1.51 (P > 0.1)	−1.56 (0.1 > P > 0.05)
COI, Clade B	122	15	0.67	0.0018	11	0.42	−2.36 (P < 0.05)	−2.05 (P < 0.05)
COI, Clade D	541	12	0.51	0.0014	12	0.45	−1.75 (P > 0.1)	−1.55 (0.1 > P > 0.05)
Cytb, all	479	52	15.48	0.066	23	0.75	0.66 (P > 0.1)	2.52 (P < 0.05)
Cytb, Clade A	225	9	0.47	0.0020	10	0.37	−1.25 (P > 0.1)	−1.53 (0.1 > P > 0.05)
Cytb, Clade B	108	5	0.34	0.0015	6	0.28	−3.26 (P < 0.05)	−1.35 (P > 0.1)
Cytb, Clade C	5	*	*	*	*	*	—	*
Cytb, Clade D	136	6	1.12	0.0038	6	0.544	−1.88 (P > 0.1)	0.049 (P > 0.1)
Cytb, Clade E	5	2	0.80	0.0027	3	0.70	−0.97 (P > 0.1)	−0.97 (P > 0.1)

^*^Sequences show no diversity.

*n*: number of sequences; S: number of polymorphic sites; k: average number of pairwise nucleotide differences; π: nucleotide diversity; h: number of haplotypes; Hd: haplotype diversity. Tajima’s *D*: A negative Tajima’s *D* signifies an excess of low frequency polymorphisms relative to expectation. A positive Tajima’s *D* signifies low levels of both low and high frequency polymorphisms. Statistical significance: Not significant, P > 0.10.

**Table 3:** Analysis of molecular variance of COI and cytb from five mitochondrial clades of *Pediculus humanus*.

**Marker**	**Source of variation**	**Sum of squares**	**Variance components**	**Percentage of variation**	**Fixation indices**	***P*-value**
COI	Among clades	12419.35	22.38	96.62	0.966 F_CT_	<0.001
	Among populations within clades	148.52	0.38	1.63	0.480 F_SC_	<0.001
	Within populations	373.92	0.41	1.76	0.982 F_ST_	<0.001
Cytb	Among clades	3052.28	12.00	74.31	0.743 F_CT_	<0.001
	Among populations within clades	1291.02	3.71	23.00	0.895 F_SC_	<0.001
	Within populations	189.46	0.43	2.68	0.9732 F_ST_	<0.001
