# Supplementary material for: High diversity and rapid diversification in the head louse, Pediculus humanus (Pediculidae: Phthiraptera)
Source: Sci Rep. 2015 Sep 16;5:14188. doi: 10.1038/srep14188 (PMC4570997; doi:10.1038/srep14188)
Supplement: Supplementary Information [file srep14188-s1.pdf]

High diversity and rapid diversification in the head louse, *Pediculus humanus* (Pediculidae: Phthiraptera)

Muhammad Ashfaq[1], Sean Prosser[1], Saima Nasir[2], Mariyam Masood[3], Sujeevan Ratnasingham[1], Paul D. N. Hebert[1]

[1] Biodiversity Institute of Ontario, University of Guelph, Guelph, ON, Canada

[2] Pakistan Council for Science and Technology, Islamabad, Pakistan

[3] National Institute for Biotechnology and Genetic Engineering, Jhang Road, Faisalabad, Pakistan

Supplementary figure legends (figures follow in the order of the legends)

Fig. S1. Maximum parsimony analysis of COI (A) and cytb (B) sequences for head lice clades. Bootstrap values (500 replicates) are shown next to the branches.

Fig. S2. Phylogenetic tree of COI (A) and cytb (B) sequences for head lice clades from Bayesian inference. Posterior probabilities are indicated at nodes.

Fig. S3. Amino acid alignment of COI (A) and cytb (B) sequences from *Homo* and *Pan* species (upper panel) and their lice (lower panel). Amino acid differences are boxed.

Table S1. GPS coordinates of lice sampling locations.

Table S2. Accessions from GenBank and from this study included in the analysis.

Suppl. 1. COI and cytb based species delimitation using the online version of PTP (http://species.h-its.org/ptp/).

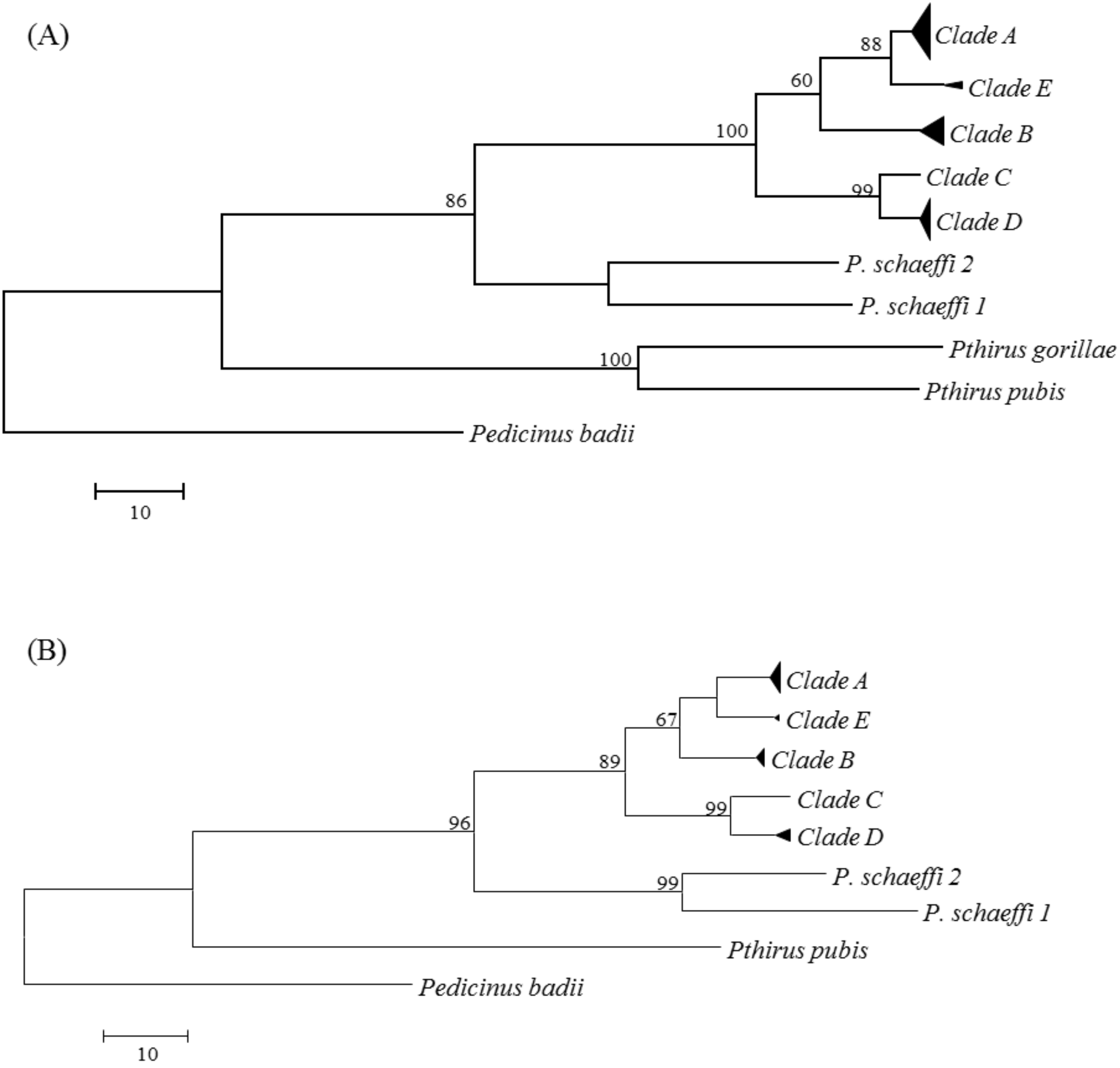

(A)
88
Clade A
60
Clade E
100
Clade B
Clade C
99
Clade D
86
P. schaeffi 2
P. schaeffi 1
100
Pthirus gorillae
Pthirus pubis
Pedicinus badii
10
(B)
Clade A
67
Clade E
89
Clade B
Clade C
99
96
Clade D
99
P. schaeffi 2
P. schaeffi 1
Pthirus pubis
Pedicinus badii
10

(A)

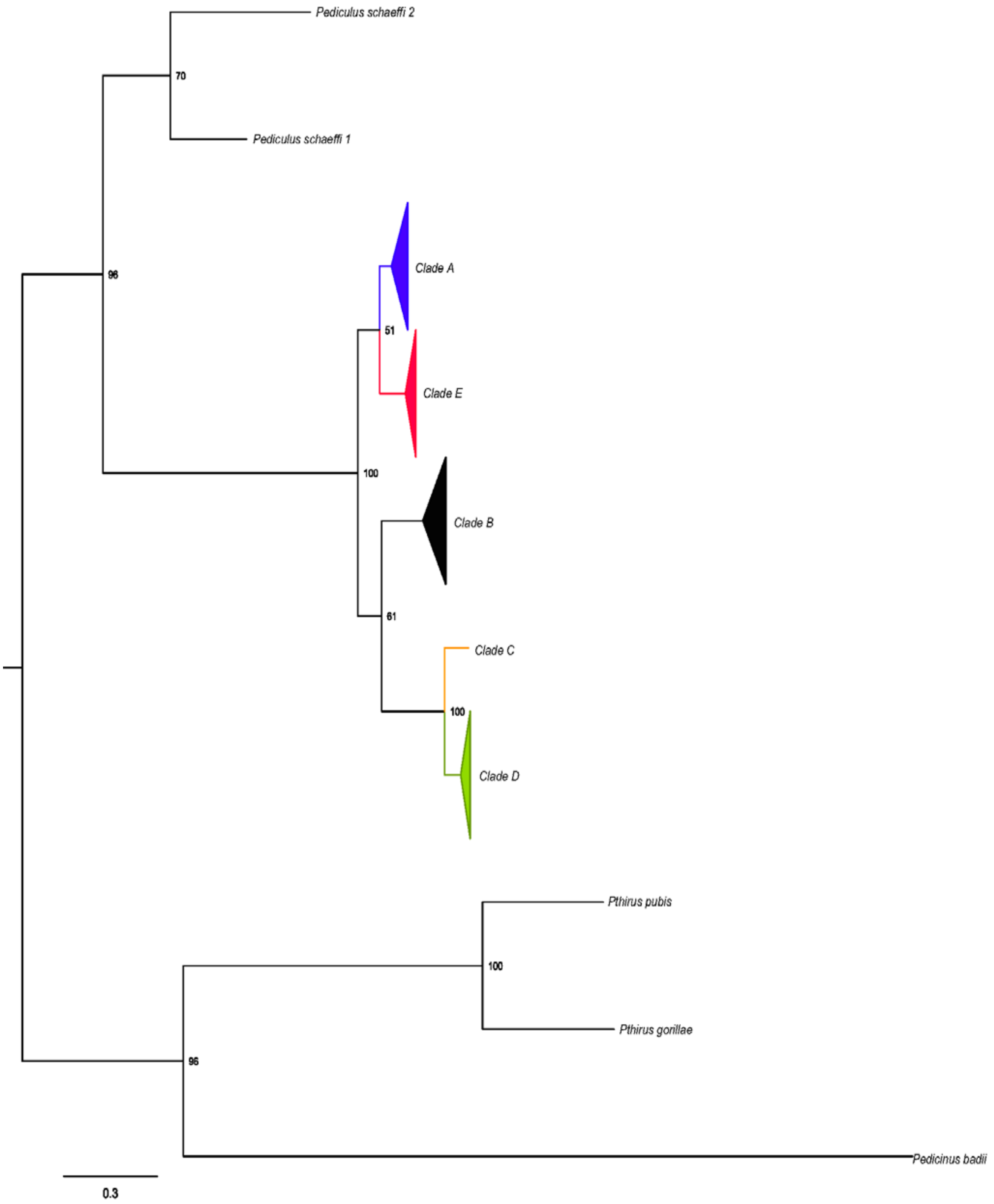

Pediculus schaeffi 2
70
Pediculus schaeffi 1
96
Clade A
51
Clade E
100
Clade B
61
Clade C
100
Clade D
Pthirus pubis
100
Pthirus gorillae
96
Pedicinus badii
0.3

(B)

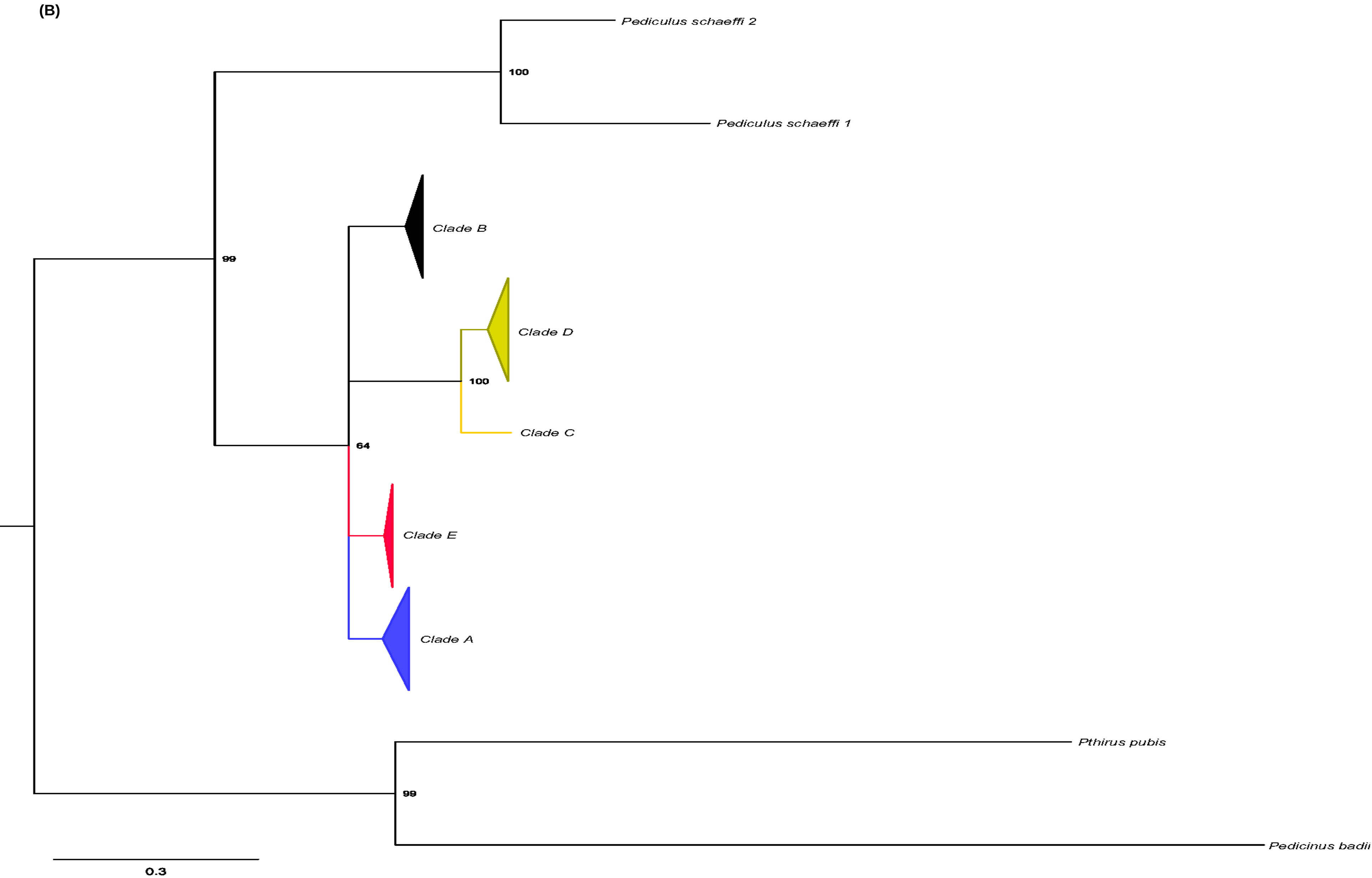

Pediculus schaeffi 2
100
Pediculus schaeffi 1
99
Clade B
Clade D
100
Clade C
64
Clade E
Clade A
Pthirus pubis
99
Pedicinus badii
0.3

(A)

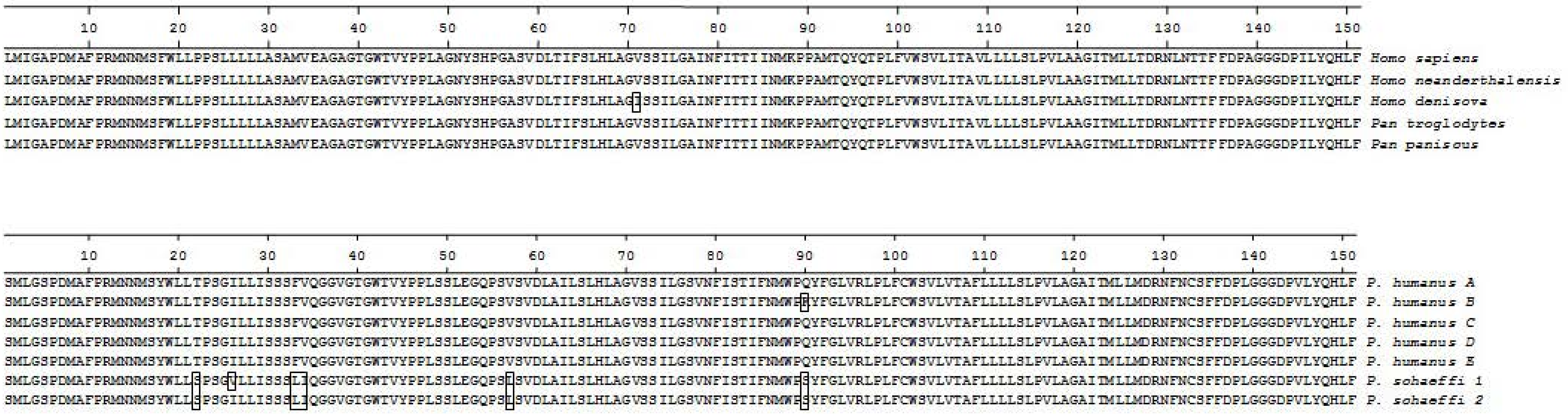
Homo sapiens
Homo neanderthalensis
Homo denisova
Pan troglodytes
Pan paniscus
P. humanus A
P. humanus B
P. humanus C
P. humanus D
P. humanus E
P. schaeffi 1
P. schaeffi 2

(B)

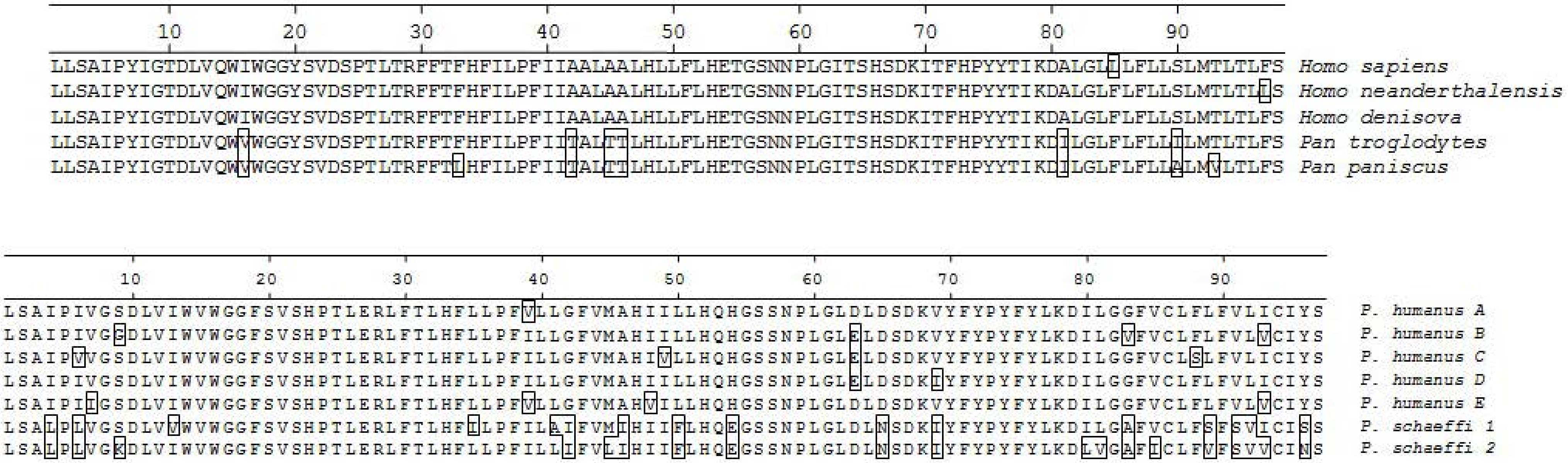
Homo sapiens
Homo neanderthalensis
Homo denisova
Pan troglodytes
Pan paniscus
P. humanus A
P. humanus B
P. humanus C
P. humanus D
P. humanus E
P. schaeffi 1
P. schaeffi 2

Table S1: Cities and GPS coordinates of lice sampling locations

Country	City	Latitude	Longitude
Pakistan	Bhakkar	31.623	71.061
		31.624	71.072
		31.611	71.062
		31.607	71.083
		31.64	71.075
		31.635	71.059
		31.603	71.059
	Dargae	34.504	71.905
	Faisalabad	31.3833	73.0167
		31.406	73.033
		31.407	73.036
		31.437	72.968
		31.4	73.035
		31.37	72.936
		31.368	72.941
		31.369	72.942
		31.417	73.018
		31.419	73.02
		31.411	73.025
		31.41	73.022
		31.402	73.018
		31.403	73.027
		31.407	73.015
		31.386	73.013
		31.381	73.018
		31.395	73.025
		31.396	73.025
		31.38	73.019
		31.407	73.043
		31.408	73.044
		31.407	73.042
		31.407	73.041
		31.406	73.042
		31.406	73.041
		31.405	73.042
		31.405	73.043
		31.404	73.043
		31.405	73.044
		31.402	73.033
		31.403	73.035
		31.403	73.033
		31.402	73.032
		31.403	73.036
		31.402	73.035
		31.403	73.031

		31.401	73.031
		31.371	73.036
		31.371	73.035
		31.372	73.035
		31.22	73.024
		31.051	72.555
		31.409	73.032
		31.41	73.03
	Islamabad	33.65	73.05
		33.6333	73.1
		33.65	73.0833
		33.5667	73.1833
		33.6333	72.95
		33.5333	73.15
		33.633	73.1
		33.633	73.033
		33.567	73.183
		33.633	72.95
		33.533	73.15
		33.65	73.083
	Lahore	31.478	74.327
		31.57	74.321
		31.514	74.284
		31.514	74.294
		31.501	74.365
	Multan	29.879	71.298
		30.195	71.458
		30.212	71.444
	Rawalpindi	33.5833	73.1
		33.5833	73.0833
		33.6	73.0667
		33.65	73.0833
		33.583	73.1
		33.583	73.083
		33.6	73.067
		33.65	73.083
	Sargodha	32.069	72.68
		32.105	72.713
		32.089	72.678
	Swat	34.305	71.544
		34.304	71.542
		34.302	71.541
		34.303	71.551
Canada	Guelph	43.31	-80.14
Egypt	Elshatby	31.206	29.92
Honduras	Buenos Aires	15.497	-88.188
South Africa	Johannesburg	-28.017	28.4

Table S2. Accessions from Genbank and from this study included in the analysis

Genbank				This study			
**COI**	**Country**	**CytB**	**Country**	**COI**	**Country**	**CytB**	**Country**
AY239285	Zimbabwe	AY316753	United Kingdom	KJ840310	Pakistan	KJ840550	South Africa
AY239286	Russia	AY316754	Taiwan	KJ840146	Pakistan	KJ840551	Pakistan
AY239287	France	AY316755	Laos	KJ839972	Pakistan	KJ840552	South Africa
AY239288	Portugal	AY316756	Papua New Guinea	KJ839948	Pakistan	KJ840553	Egypt
AY316748	USA	AY316757	Papua New Guinea	KJ840132	Pakistan	KJ840554	South Africa
AY316749	Ecuador	AY316758	Philippines	KJ840358	Pakistan	KJ840555	Egypt
AY316750	United Kingdom	AY316759	Ecuador	KJ840383	Pakistan	KJ840556	Pakistan
AY316751	Germany	AY316760	Panama	KJ840037	Pakistan	KJ840557	Egypt
AY316752	Ethiopia	AY316761	Germany	KJ840157	Pakistan	KJ840559	Egypt
AY589944	Nepal	AY316762	Germany	KJ840267	Pakistan	KJ840560	Egypt
AY589945	Nepal	AY316763	Germany	KJ839930	Pakistan	KJ840561	Egypt
AY589946	Nepal	AY316764	Germany	KJ840493	Pakistan	KJ840562	Egypt
AY589947	Nepal	AY316765	Ethiopia	KJ840306	Pakistan	KJ840563	Pakistan
AY589948	Nepal	AY316766	Ethiopia	KJ840039	Pakistan	KJ840564	Pakistan
AY589949	Nepal	AY316767	Ethiopia	KJ839858	Pakistan	KJ840565	Pakistan
AY589950	Nepal	AY316768	Ethiopia	KJ840426	Pakistan	KJ840566	Pakistan
AY589951	Nepal	AY316769	Ethiopia	KJ840322	Pakistan	KJ840567	Pakistan
AY589952	Nepal	AY316770	Ethiopia	KJ840109	Pakistan	KJ840568	Egypt
AY589953	Nepal	AY316771	Nepal	KJ840069	Pakistan	KJ840569	Pakistan
AY589954	Nepal	AY316772	Nepal	KJ840308	Pakistan	KJ840570	Pakistan
AY589955	Nepal	AY316773	Ethiopia	KJ840468	Pakistan	KJ840571	Pakistan
AY589956	Nepal	AY316774	Ethiopia	KJ840506	Pakistan	KJ840572	Egypt
AY589957	Nepal	AY316775	Ethiopia	KJ840140	Pakistan	KJ840573	Pakistan
AY589958	Nepal	AY316776	Germany	KJ840473	Pakistan	KJ840574	Egypt
AY589959	Nepal	AY316777	United Kingdom	KJ840092	Pakistan	KJ840575	Pakistan
AY589960	Nepal	AY316778	United Kingdom	KJ839938	Egypt	KJ840576	Egypt
AY589961	Nepal	AY316779	Germany	KJ840417	Egypt	KJ840577	South Africa
AY589962	Nepal	AY316780	Germany	KJ840416	Egypt	KJ840578	Pakistan
AY589963	Nepal	AY316781	Iran	KJ840377	Egypt	KJ840579	Pakistan
AY589964	Nepal	AY316782	Iran	KJ839873	Egypt	KJ840580	South Africa
AY589965	Nepal	AY316783	Germany	KJ840494	Egypt	KJ840581	Pakistan
AY589966	China	AY316784	Ethiopia	KJ840178	Egypt	KJ840582	South Africa
AY589967	Nepal	AY316785	Ethiopia	KJ840366	Egypt	KJ840583	Pakistan
AY589968	Nepal	AY316786	Ethiopia	KJ840027	Egypt	KJ840584	Egypt
AY589969	Nepal	AY316787	Ethiopia	KJ839927	Egypt	KJ840585	Pakistan
AY589970	China	AY316788	Ethiopia	KJ840275	Egypt	KJ840586	South Africa
AY589971	China	AY316789	Ethiopia	KJ840226	Egypt	KJ840588	Pakistan
AY589972	China	AY316790	Ethiopia	KJ840271	Egypt	KJ840589	Egypt
AY589973	Japan	AY316791	Germany	KJ840182	Egypt	KJ840590	Egypt
AY589974	China	AY316792	USA	KJ839960	Egypt	KJ840591	South Africa
AY589975	China	AY696009	USA	KJ840199	Egypt	KJ840592	Pakistan
AY589976	China	AY696010	USA	KJ840059	Egypt	KJ840593	South Africa
AY589977	China	AY696011	USA	KJ840374	Egypt	KJ840594	Egypt

AY589978	China	AY696012	Honduras	KJ839944	Egypt	KJ840595	Egypt
AY589979	Nepal	AY696013	USA	KJ840472	Egypt	KJ840596	South Africa
AY589980	Iran	AY696014	USA	KJ840031	Egypt	KJ840597	Egypt
AY589981	Iran	AY696015	Honduras	KJ840030	Egypt	KJ840598	Egypt
AY589982	Nepal	AY696016	Honduras	KJ840195	Egypt	KJ840599	Pakistan
AY589983	Nepal	AY696017	Honduras	KJ840085	Egypt	KJ840600	Pakistan
AY589984	Nepal	AY696018	Honduras	KJ840463	Egypt	KJ840601	South Africa
AY589985	Nepal	AY696019	Philippines	KJ840087	Egypt	KJ840602	Pakistan
AY589986	Nepal	AY696020	Philippines	KJ839999	Egypt	KJ840603	South Africa
AY589987	Nepal	AY696021	Philippines	KJ840460	Egypt	KJ840604	Pakistan
AY589988	Nepal	AY696022	Philippines	KJ839954	Egypt	KJ840605	Egypt
AY589989	Nepal	AY696023	Philippines	KJ840278	Egypt	KJ840606	Pakistan
AY589990	Nepal	AY696024	Philippines	KJ840093	Egypt	KJ840607	Egypt
AY589991	Nepal	AY696025	Philippines	KJ840531	Egypt	KJ840608	Egypt
AY589992	Nepal	AY696026	USA	KJ840337	Egypt	KJ840609	Egypt
AY589993	Nepal	AY696027	USA	KJ840532	Egypt	KJ840610	Egypt
AY589994	Nepal	AY696028	USA	KJ840161	Egypt	KJ840611	Egypt
AY589995	Nepal	AY696029	USA	KJ840216	Egypt	KJ840612	Pakistan
AY589996	Australia	AY696030	USA	KJ840406	Egypt	KJ840613	Pakistan
AY589997	Israel	AY696031	Canada	KJ839874	Egypt	KJ840615	Egypt
AY589998	Kenya	AY696032	Canada	KJ839857	Egypt	KJ840616	Egypt
AY589999	Papua New Guinea	AY696033	Canada	KJ840434	Egypt	KJ840617	Pakistan
AY590000	Argentina	AY696034	Canada	KJ839902	Egypt	KJ840618	Egypt
AY590001	Iran	AY696035	Canada	KJ840301	Egypt	KJ840619	Pakistan
AY590002	Iran	AY696036	Canada	KJ839876	Egypt	KJ840620	Egypt
AY590003	China	AY696037	Canada	KJ840448	Egypt	KJ840621	Egypt
AY590004	China	AY696038	USA	KJ840504	Egypt	KJ840622	South Africa
AY590005	New Zealand	AY696039	USA	KJ840290	Egypt	KJ840623	South Africa
AY590006	Australia	AY696040	USA	KJ840086	Egypt	KJ840624	Pakistan
AY590007	Australia	AY696041	USA	KJ840483	Egypt	KJ840625	Pakistan
AY590008	Australia	AY696042	Yemen	KJ840403	Egypt	KJ840626	South Africa
AY590009	China	AY696043	Philippines	KJ840393	Egypt	KJ840627	South Africa
AY590010	China	AY696044	Honduras	KJ840250	Egypt	KJ840628	Egypt
AY590011	China	AY696045	Philippines	KJ840166	Egypt	KJ840629	Pakistan
AY590012	Nepal	AY696046	Philippines	KJ839868	Egypt	KJ840630	Pakistan
AY590013	Nepal	AY696047	USA	KJ840078	Egypt	KJ840631	South Africa
AY590014	France	AY696048	USA	KJ840004	Egypt	KJ840632	Egypt
AY590015	China	AY696049	USA	KJ840124	Egypt	KJ840633	South Africa
AY590016	China	AY696050	Papua New Guinea	KJ839986	Egypt	KJ840634	Pakistan
AY590017	Nepal	AY696051	Papua New Guinea	KJ840204	Egypt	KJ840635	Pakistan
AY590018	China	AY696052	Papua New Guinea	KJ839905	Egypt	KJ840636	Pakistan
AY590019	China	AY696053	USA	KJ840219	Egypt	KJ840637	Pakistan
AY590020	China	AY696054	USA	KJ840501	Egypt	KJ840638	Pakistan
AY590021	China	AY696055	USA	KJ839939	Egypt	KJ840639	Pakistan
AY590022	China	AY696056	USA	KJ839998	Egypt	KJ840640	Egypt
AY590023	Israel	AY696057	Philippines	KJ840020	Egypt	KJ840641	Egypt
AY590024	Australia	AY696058	Philippines	KJ840252	Egypt	KJ840642	Egypt

AY590025	Australia	AY696059	Honduras	KJ840423	Egypt	KJ840643	Egypt
AY590026	Australia	AY696060	Honduras	KJ839870	Egypt	KJ840644	Egypt
AY590027	Australia	AY696061	Philippines	KJ840213	Egypt	KJ840645	South Africa
AY590028	Australia	AY696062	Philippines	KJ839899	Egypt	KJ840646	South Africa
AY590029	Australia	AY696063	Papua New Guinea	KJ840340	Egypt	KJ840647	South Africa
AY590030	France	AY696064	Papua New Guinea	KJ840299	Egypt	KJ840648	Egypt
AY590031	Argentina	AY696065	Papua New Guinea	KJ840349	Egypt	KJ840649	Egypt
AY590032	Brazil	AY696066	Papua New Guinea	KJ840048	Egypt	KJ840650	South Africa
AY590033	Brazil	EF653430	Peru	KJ839892	Egypt	KJ840651	Pakistan
AY590034	Nepal	FJ267428	Unknown	KJ839877	Egypt	KJ840652	South Africa
AY590035	Nepal	FJ267429	Unknown	KJ840129	Egypt	KJ840653	Pakistan
AY590036	Nepal	FJ267430	Unknown	KJ840000	Egypt	KJ840654	South Africa
AY590037	Nepal	FJ267431	Unknown	KJ839946	Egypt	KJ840655	Egypt
AY590038	Nepal	FJ267432	Unknown	KJ840247	Egypt	KJ840656	South Africa
AY590039	Nepal	FJ267433	Unknown	KJ840356	Egypt	KJ840657	Honduras
AY590040	Nepal	FJ499475	Unknown	KJ840474	Egypt	KJ840658	Pakistan
AY590041	Nepal	GU323324	Russia	KJ840487	Canada	KJ840659	Egypt
AY695940	USA	GU323325	Mexico	KJ840294	South Africa	KJ840660	South Africa
AY695941	USA	GU323326	Russia	KJ840014	South Africa	KJ840661	Egypt
AY695942	USA	GU323327	Unknown	KJ840492	South Africa	KJ840662	Pakistan
AY695943	USA	GU323328	Russia	KJ840436	South Africa	KJ840663	Egypt
AY695944	Honduras	GU323329	Russia	KJ840328	South Africa	KJ840664	Pakistan
AY695945	USA	GU323330	USA	KJ840344	South Africa	KJ840665	South Africa
AY695946	USA	GU323331	Mexico	KJ840046	South Africa	KJ840666	Pakistan
AY695947	Honduras	GU323332	USA	KJ840447	South Africa	KJ840667	Pakistan
AY695948	Honduras	GU323333	USA	KJ840033	South Africa	KJ840668	Egypt
AY695949	Honduras	GU323334	France	KJ840303	South Africa	KJ840669	Egypt
AY695950	Honduras	JF694384	Ethiopia	KJ840348	South Africa	KJ840670	Pakistan
AY695951	Philippines	JF694385	Ethiopia	KJ840549	South Africa	KJ840671	Egypt
AY695952	Philippines	JF694386	Ethiopia	KJ840259	South Africa	KJ840673	Pakistan
AY695953	Philippines	JF694387	Ethiopia	KJ840117	South Africa	KJ840674	South Africa
AY695954	Philippines	JF694388	Ethiopia	KJ840160	South Africa	KJ840675	Egypt
AY695955	Philippines	JF694389	Ethiopia	KJ840336	South Africa	KJ840676	Pakistan
AY695956	Philippines	JF694390	Ethiopia	KJ840482	South Africa	KJ840677	South Africa
AY695957	Philippines	JF694391	Ethiopia	KJ839931	South Africa	KJ840678	Egypt
AY695958	USA	JF694392	Ethiopia	KJ839846	South Africa	KJ840679	Pakistan
AY695959	USA	JF694393	Ethiopia	KJ840341	South Africa	KJ840680	Egypt
AY695960	USA	JF694394	Ethiopia	KJ840422	South Africa	KJ840681	Pakistan
AY695961	USA	JF694395	Ethiopia	KJ840350	South Africa	KJ840682	Pakistan
AY695962	USA	JF694396	Ethiopia	KJ840288	South Africa	KJ840683	Egypt
AY695963	Canada	JF694397	Ethiopia	KJ840060	South Africa	KJ840684	Pakistan
AY695964	Canada	JF694398	Ethiopia	KJ839949	South Africa	KJ840685	Pakistan
AY695965	Canada	JF694399	Ethiopia	KJ840103	South Africa	KJ840686	South Africa
AY695966	Canada	JF694400	Ethiopia	KJ840141	South Africa	KJ840687	South Africa
AY695967	Canada	JF694401	Ethiopia	KJ840281	South Africa	KJ840688	Egypt
AY695968	Canada	JF694402	Ethiopia	KJ840169	South Africa	KJ840689	Honduras
AY695969	Canada	JF694403	Ethiopia	KJ840053	South Africa	KJ840690	Egypt

AY695970	USA	JF694404	Ethiopia	KJ840163	South Africa	KJ840691	Egypt
AY695971	USA	JF694405	Ethiopia	KJ840036	South Africa	KJ840692	Pakistan
AY695972	USA	JF694406	Ethiopia	KJ840499	South Africa	KJ840693	Egypt
AY695973	USA	JF694407	Ethiopia	KJ840188	South Africa	KJ840694	South Africa
AY695974	Yemen	JF694408	Ethiopia	KJ839977	South Africa	KJ840695	South Africa
AY695975	Philippines	JF694409	Ethiopia	KJ840097	South Africa	KJ840696	Egypt
AY695976	Honduras	JF694410	Ethiopia	KJ840206	South Africa	KJ840697	Pakistan
AY695977	Philippines	JF694411	Ethiopia	KJ840300	South Africa	KJ840698	Egypt
AY695978	Philippines	JF694412	Ethiopia	KJ840243	South Africa	KJ840699	Egypt
AY695979	USA	JF694413	Ethiopia	KJ839921	South Africa	KJ840700	Egypt
AY695980	USA	JF694414	Ethiopia	KJ840236	South Africa	KJ840701	South Africa
AY695981	USA	JF694415	Ethiopia	KJ839860	South Africa	KJ840702	Pakistan
AY695982	Papua New Guinea	JF694416	Ethiopia	KJ839936	South Africa	KJ840703	South Africa
AY695983	Papua New Guinea	JF694417	Ethiopia	KJ840043	South Africa	KJ840704	Egypt
AY695984	Papua New Guinea	JF694418	Ethiopia	KJ840152	South Africa	KJ840705	Pakistan
AY695985	USA	JF694419	Ethiopia	KJ840249	Honduras	KJ840706	South Africa
AY695986	USA	JF694420	Ethiopia	KJ840164	Honduras	KJ840707	South Africa
AY695987	USA	JF694421	Ethiopia	KJ840331	Honduras	KJ840708	Pakistan
AY695988	USA	JF694422	Ethiopia	KJ839933	Pakistan	KJ840709	Egypt
AY695989	Philippines	JF694423	Ethiopia	KJ840321	Pakistan	KJ840710	South Africa
AY695990	Philippines	JF694424	Ethiopia	KJ840119	Pakistan	KJ840711	Egypt
AY695991	Honduras	JF694425	Ethiopia	KJ840343	Pakistan	KJ840712	South Africa
AY695992	Honduras	JF694426	Ethiopia	KJ840089	Pakistan	KJ840713	Pakistan
AY695993	Philippines	JF694427	Ethiopia	KJ840091	Pakistan	KJ840714	Pakistan
AY695994	Philippines	JF694428	Ethiopia	KJ840438	Pakistan	KJ840715	Pakistan
AY695995	Papua New Guinea	JF694429	Ethiopia	KJ840118	Pakistan	KJ840716	Pakistan
AY695996	Papua New Guinea	JF694430	Ethiopia	KJ840076	Pakistan	KJ840717	South Africa
AY695997	Papua New Guinea	JF694431	Ethiopia	KJ840280	Pakistan	KJ840718	Pakistan
AY695998	Papua New Guinea	JF694432	Ethiopia	KJ840158	Pakistan	KJ840719	Honduras
DQ054849	USA	JF694433	Ethiopia	KJ840329	Pakistan	KJ840720	Pakistan
EF152552	Unknown	JF694434	Ethiopia	KJ840173	Pakistan	KJ840721	South Africa
EU493361	USA	JF694435	Ethiopia	KJ840502	Pakistan	KJ840722	Egypt
EU493362	USA	JF694436	Ethiopia	KJ840421	Pakistan	KJ840723	Egypt
EU493363	USA	JF694437	Ethiopia	KJ840409	Pakistan	KJ840724	South Africa
EU493364	USA	JF694438	Ethiopia	KJ840459	Pakistan	KJ840725	South Africa
EU493365	USA	JF694439	Ethiopia	KJ840256	Pakistan	KJ840726	Egypt
EU493367	USA	JF694440	Ethiopia	KJ840251	Pakistan	KJ840727	Pakistan
EU493368	USA	JF694441	Ethiopia	KJ840006	Pakistan	KJ840728	South Africa
EU493369	USA	JF694442	Ethiopia	KJ840312	Pakistan	KJ840729	Egypt
EU493370	USA	JN400090	Senegal	KJ840127	Pakistan	KJ840730	South Africa
EU493371	USA	JN400091	Senegal	KJ839880	Pakistan	KJ840731	Pakistan
EU493372	USA	JN400092	Senegal	KJ840022	Pakistan	KJ840732	Pakistan
EU493373	Panama	JN969581	Senegal	KJ839973	Pakistan	KJ840733	Pakistan
EU493374	Panama	JN969582	Senegal	KJ840070	Pakistan	KJ840734	Egypt
EU493375	USA	JN969583	Senegal	KJ840414	Pakistan	KJ840735	Pakistan
EU493376	USA	JN969584	Senegal	KJ839955	Pakistan	KJ840736	Pakistan
EU493377	USA	JN969585	Senegal	KJ839967	Pakistan	KJ840737	Egypt

EU493378	USA	JN969586	Senegal	KJ840056	Pakistan	KJ840738	Pakistan
EU493379	USA	JN969587	Senegal	KJ839978	Pakistan	KJ840739	Egypt
EU493380	USA	JN969588	Senegal	KJ839920	Pakistan	KJ840740	Egypt
EU493381	USA	JX080399	Unknown	KJ840305	Pakistan	KJ840742	Pakistan
EU493382	USA	KC685772	Unknown	KJ840057	Pakistan	KJ840743	Pakistan
EU493383	Argentina	KC685773	Unknown	KJ839896	Pakistan	KJ840744	Egypt
EU493384	Papua New Guinea	KC685774	Unknown	KJ839923	Pakistan	KJ840745	Honduras
EU493385	Papua New Guinea	KC685775	Unknown	KJ840008	Pakistan	KJ840746	Pakistan
EU493386	Norway	KC685776	Unknown	KJ840068	Pakistan	KJ840747	Egypt
EU493387	Norway	KC685777	Unknown	KJ840225	Pakistan	KJ840748	Egypt
EU493388	Colombia	KC685778	Unknown	KJ839871	Pakistan	KJ840749	Egypt
EU493389	Colombia	KC685779	Unknown	KJ839928	Pakistan	KJ840750	Egypt
EU493390	Ecuador	KC685780	Unknown	KJ840428	Pakistan	KJ840751	Pakistan
EU493391	Ecuador	KC685781	Unknown	KJ840295	Pakistan	KJ840752	Pakistan
EU493392	Cook Islands	KC685782	Unknown	KJ839845	Pakistan	KJ840753	South Africa
EU493393	Mongolia	KC685783	Unknown	KJ839958	Pakistan	KJ840754	Egypt
EU493394	Burundi	KC685784	Unknown	KJ839904	Pakistan	KJ840755	Pakistan
EU493395	Burundi	KC685785	Unknown	KJ840527	Pakistan	KJ840756	Egypt
EU493396	United Kingdom	KC685786	Unknown	KJ839907	Pakistan	KJ840757	South Africa
EU493397	United Kingdom	KC685787	Unknown	KJ840505	Pakistan	KJ840758	South Africa
EU493398	United Kingdom	KC685788	Unknown	KJ840245	Pakistan	KJ840759	Egypt
EU493399	United Kingdom	KC685789	Unknown	KJ840307	Pakistan	KJ840760	Pakistan
EU493400	United Kingdom	KC685790	Unknown	KJ839901	Pakistan	KJ840761	Pakistan
EU493401	United Kingdom	KC685791	Unknown	KJ840175	Pakistan	KJ840762	South Africa
EU493402	United Kingdom	KC867681	Unknown	KJ839859	Pakistan	KJ840763	Egypt
EU493403	United Kingdom	KC867682	Unknown	KJ840538	Pakistan	KJ840764	Egypt
EU493404	United Kingdom	KC867683	Unknown	KJ840311	Pakistan	KJ840765	Egypt
EU493405	United Kingdom	KF498962	Chile	KJ840121	Pakistan	KJ840766	Pakistan
EU493406	United Kingdom	KF498963	Chile	KJ840246	Pakistan	KJ840767	South Africa
EU493407	United Kingdom	KF693771	Algeria	KJ840196	Pakistan	KJ840768	Egypt
EU493408	United Kingdom	KF693772	Algeria	KJ839916	Pakistan	KJ840769	South Africa
EU493409	United Kingdom	KF693773	Algeria	KJ840360	Pakistan	KJ840770	Egypt
EU493410	United Kingdom	KF693774	Algeria	KJ840529	Pakistan	KJ840771	South Africa
EU493411	United Kingdom	KF693775	Algeria	KJ839847	Pakistan	KJ840772	Egypt
EU493412	United Kingdom	KF700675	Algeria	KJ839910	Pakistan	KJ840773	South Africa
EU493413	United Kingdom	KF700676	Algeria	KJ840273	Pakistan	KJ840774	South Africa
EU493414	United Kingdom	KF700677	Algeria	KJ839987	Pakistan	KJ840775	Egypt
EU493415	United Kingdom	KF700678	Algeria	KJ840476	Pakistan	KJ840776	Egypt
EU493416	United Kingdom	KF700679	Algeria	KJ839981	Pakistan	KJ840777	South Africa
EU493417	United Kingdom	KF700680	Algeria	KJ840177	Pakistan	KJ840778	Egypt
EU493418	United Kingdom	KF700681	Algeria	KJ840035	Pakistan	KJ840779	Egypt
EU493419	United Kingdom	KF700682	Algeria	KJ840098	Pakistan	KJ840780	Pakistan
EU493420	United Kingdom	KF700683	Algeria	KJ839884	Pakistan	KJ840781	Egypt
EU493421	United Kingdom	KF700684	Algeria	KJ840452	Pakistan	KJ840782	South Africa
EU493422	United Kingdom	KF700685	Algeria	KJ840051	Pakistan	KJ840783	Pakistan
EU493423	United Kingdom	KF700686	Algeria	KJ840491	Pakistan	KJ840784	Pakistan
EU493424	USA	KF700687	Algeria	KJ839919	Pakistan	KJ840785	Pakistan

EU493425	USA
EU493426	USA
EU493427	USA
EU493428	USA
EU493429	USA
EU493430	USA
EU493431	USA
EU493432	USA
EU493433	USA
EU493434	USA
EU493435	USA
EU493436	USA
EU493437	USA
EU493438	USA
EU493439	USA
EU493440	USA
EU493441	USA
EU493442	USA
EU493443	United Kingdom
EU493444	United Kingdom
EU493445	USA
EU493446	USA
EU493447	USA
FJ267425	Unknown
FJ267426	Unknown
FJ499477	Unknown
JX080396	Unknown
KC685849	Unknown
HM357241	Australia
KC685838	Unknown
KC685839	Unknown
KC685840	Unknown
KC685841	Unknown
KC685842	Unknown
KC685845	Unknown
KC685846	Unknown
KF700688	Algeria
KF700689	Algeria
KF700690	Algeria
KF700691	Algeria
KF700692	Algeria
KF700693	Algeria
KF700694	Algeria
KF700695	Algeria
FJ821011	USA
KJ840396	Pakistan
KJ840412	Pakistan
KJ840379	Pakistan
KJ840272	Pakistan
KJ839878	Pakistan
KJ840380	Pakistan
KJ840439	Pakistan
KJ840010	Pakistan
KJ840530	Pakistan
KJ839866	Pakistan
KJ840498	Pakistan
KJ840526	Pakistan
KJ840368	Pakistan
KJ839875	Pakistan
KJ839895	Pakistan
KJ840413	Pakistan
KJ840330	Pakistan
KJ840449	Pakistan
KJ839911	Pakistan
KJ839966	Pakistan
KJ840017	Pakistan
KJ840005	Pakistan
KJ839852	Pakistan
KJ840372	Pakistan
KJ840324	Pakistan
KJ840186	Pakistan
KJ840207	Pakistan
KJ840345	Pakistan
KJ840181	Pakistan
KJ840139	Pakistan
KJ840165	Pakistan
KJ840523	Pakistan
KJ840435	Pakistan
KJ840440	Pakistan
KJ839926	Pakistan
KJ840123	Pakistan
KJ840090	Pakistan
KJ840234	Pakistan
KJ840394	Pakistan
KJ839863	Pakistan
KJ840240	Pakistan
KJ840454	Pakistan
KJ840208	Pakistan
KJ839872	Pakistan
KJ840094	Pakistan
KJ840539	Pakistan
KJ840378	Pakistan
KJ840786	Pakistan
KJ840787	Egypt
KJ840788	Pakistan
KJ840790	Egypt
KJ840791	Pakistan
KJ840792	Pakistan
KJ840793	Pakistan
KJ840795	South Africa

KJ839952	Pakistan
KJ840102	Pakistan
KJ840154	Pakistan
KJ840001	Pakistan
KJ840248	Pakistan
KJ840296	Pakistan
KJ840083	Pakistan
KJ840401	Pakistan
KJ840064	Pakistan
KJ840485	Pakistan
KJ840544	Pakistan
KJ840334	Pakistan
KJ840067	Pakistan
KJ840471	Pakistan
KJ840212	Pakistan
KJ840257	Pakistan
KJ840191	Pakistan
KJ840197	Pakistan
KJ839988	Pakistan
KJ840228	Pakistan
KJ840104	Pakistan
KJ840049	Pakistan
KJ840218	Pakistan
KJ840442	Pakistan
KJ839848	Pakistan
KJ839983	Pakistan
KJ839970	Pakistan
KJ840170	Pakistan
KJ839932	Pakistan
KJ840239	Pakistan
KJ840319	Pakistan
KJ840369	Pakistan
KJ839969	Pakistan
KJ839997	Pakistan
KJ840479	Pakistan
KJ840079	Pakistan
KJ840171	Pakistan
KJ840437	Pakistan
KJ840387	Pakistan
KJ840144	Pakistan
KJ840443	Pakistan
KJ840318	Pakistan
KJ839929	Pakistan
KJ840106	Pakistan
KJ840162	Pakistan
KJ840400	Pakistan
KJ840277	Pakistan

KJ840325	Pakistan
KJ839838	Pakistan
KJ840179	Pakistan
KJ840397	Pakistan
KJ839913	Pakistan
KJ840433	Pakistan
KJ840114	Pakistan
KJ839957	Pakistan
KJ840332	Pakistan
KJ840516	Pakistan
KJ840007	Pakistan
KJ840183	Pakistan
KJ840342	Pakistan
KJ840430	Pakistan
KJ840233	Pakistan
KJ840327	Pakistan
KJ840009	Pakistan
KJ840222	Pakistan
KJ839864	Pakistan
KJ840074	Pakistan
KJ840385	Pakistan
KJ840266	Pakistan
KJ839840	Pakistan
KJ840231	Pakistan
KJ840364	Pakistan
KJ840238	Pakistan
KJ840202	Pakistan
KJ840445	Pakistan
KJ840381	Pakistan
KJ840016	Pakistan
KJ840003	Pakistan
KJ840507	Pakistan
KJ840223	Pakistan
KJ840077	Pakistan
KJ840187	Pakistan
KJ840285	Pakistan
KJ840073	Pakistan
KJ840054	Pakistan
KJ839909	Pakistan
KJ840547	Pakistan
KJ840450	Pakistan
KJ840451	Pakistan
KJ840242	Pakistan
KJ840235	Pakistan
KJ840082	Pakistan
KJ840522	Pakistan
KJ840002	Pakistan

KJ839942	Pakistan
KJ840384	Pakistan
KJ840469	Pakistan
KJ840167	Pakistan
KJ840040	Pakistan
KJ839953	Pakistan
KJ840386	Pakistan
KJ840230	Pakistan
KJ840269	Pakistan
KJ840080	Pakistan
KJ840055	Pakistan
KJ840072	Pakistan
KJ840512	Pakistan
KJ840420	Pakistan
KJ840405	Pakistan
KJ840122	Pakistan
KJ840411	Pakistan
KJ840112	Pakistan
KJ840024	Pakistan
KJ839903	Pakistan
KJ839861	Pakistan
KJ840359	Pakistan
KJ840425	Pakistan
KJ840176	Pakistan
KJ840317	Pakistan
KJ840172	Pakistan
KJ840481	Pakistan
KJ840390	Pakistan
KJ839881	Pakistan
KJ840028	Pakistan
KJ840466	Pakistan
KJ839976	Pakistan
KJ840464	Pakistan
KJ840338	Pakistan
KJ839865	Pakistan
KJ840138	Pakistan
KJ840265	Pakistan
KJ840339	Pakistan
KJ840540	Pakistan
KJ840478	Pakistan
KJ840038	Pakistan
KJ839934	Pakistan
KJ840497	Pakistan
KJ839912	Pakistan
KJ840404	Pakistan
KJ840419	Pakistan
KJ840011	Pakistan

KJ839937	Pakistan
KJ840535	Pakistan
KJ839965	Pakistan
KJ839974	Pakistan
KJ840244	Pakistan
KJ840018	Pakistan
KJ840151	Pakistan
KJ840289	Pakistan
KJ840254	Pakistan
KJ839943	Pakistan
KJ840347	Pakistan
KJ840066	Pakistan
KJ839947	Pakistan
KJ840282	Pakistan
KJ840062	Pakistan
KJ840147	Pakistan
KJ840541	Pakistan
KJ840220	Pakistan
KJ840135	Pakistan
KJ840335	Pakistan
KJ840211	Pakistan
KJ839993	Pakistan
KJ840260	Pakistan
KJ840012	Pakistan
KJ840192	Pakistan
KJ839889	Pakistan
KJ840284	Pakistan
KJ839917	Pakistan
KJ840237	Pakistan
KJ840155	Pakistan
KJ840488	Pakistan
KJ840304	Pakistan
KJ840131	Pakistan
KJ840025	Pakistan
KJ840227	Pakistan
KJ840444	Pakistan
KJ839898	Pakistan
KJ840110	Pakistan
KJ840357	Pakistan
KJ839935	Pakistan
KJ840286	Pakistan
KJ840019	Pakistan
KJ840457	Pakistan
KJ840533	Pakistan
KJ840462	Pakistan
KJ840185	Pakistan
KJ840276	Pakistan

KJ840407	Pakistan
KJ840107	Pakistan
KJ840258	Pakistan
KJ840520	Pakistan
KJ840500	Pakistan
KJ839842	Pakistan
KJ839985	Pakistan
KJ840115	Pakistan
KJ840029	Pakistan
KJ840355	Pakistan
KJ839924	Pakistan
KJ840528	Pakistan
KJ839950	Pakistan
KJ840232	Pakistan
KJ839991	Pakistan
KJ839989	Pakistan
KJ840209	Pakistan
KJ840026	Pakistan
KJ840376	Pakistan
KJ840302	Pakistan
KJ839839	Pakistan
KJ840099	Pakistan
KJ840261	Pakistan
KJ840508	Pakistan
KJ840021	Pakistan
KJ840453	Pakistan
KJ839890	Pakistan
KJ840013	Pakistan
KJ840524	Pakistan
KJ839908	Pakistan
KJ840189	Pakistan
KJ840255	Pakistan
KJ839975	Pakistan
KJ839982	Pakistan
KJ840263	Pakistan
KJ839925	Pakistan
KJ840313	Pakistan
KJ840217	Pakistan
KJ839914	Pakistan
KJ840431	Pakistan
KJ839941	Pakistan
KJ839915	Pakistan
KJ840352	Pakistan
KJ840137	Pakistan
KJ840461	Pakistan
KJ839992	Pakistan
KJ840105	Pakistan

KJ840537	Pakistan
KJ840052	Pakistan
KJ840418	Pakistan
KJ840511	Pakistan
KJ840042	Pakistan
KJ839867	Pakistan
KJ840410	Pakistan
KJ840071	Pakistan
KJ840388	Pakistan
KJ839918	Pakistan
KJ840542	Pakistan
KJ839869	Pakistan
KJ840514	Pakistan
KJ840361	Pakistan
KJ839980	Pakistan
KJ839855	Pakistan
KJ839891	Pakistan
KJ840432	Pakistan
KJ840270	Pakistan
KJ840467	Pakistan
KJ840408	Pakistan
KJ840293	Pakistan
KJ840484	Pakistan
KJ839979	Pakistan
KJ840513	Pakistan
KJ840111	Pakistan
KJ839906	Pakistan
KJ840346	Pakistan
KJ840061	Pakistan
KJ840128	Pakistan
KJ840198	Pakistan
KJ840120	Pakistan
KJ840519	Pakistan
KJ840134	Pakistan
KJ840375	Pakistan
KJ839922	Pakistan
KJ840145	Pakistan
KJ840333	Pakistan
KJ839883	Pakistan
KJ839853	Pakistan
KJ840287	Pakistan
KJ840315	Pakistan
KJ839959	Pakistan
KJ840253	Pakistan
KJ840214	Pakistan
KJ840142	Pakistan
KJ840316	Pakistan

KJ840095	Pakistan
KJ840133	Pakistan
KJ840168	Pakistan
KJ840362	Pakistan
KJ840047	Pakistan
KJ840044	Pakistan
KJ839849	Pakistan
KJ839882	Pakistan
KJ839844	Pakistan
KJ840084	Pakistan
KJ840521	Pakistan
KJ840455	Pakistan
KJ840297	Pakistan
KJ840424	Pakistan
KJ840292	Pakistan
KJ839990	Pakistan
KJ840509	Pakistan
KJ840370	Pakistan
KJ840291	Pakistan
KJ840465	Pakistan
KJ840415	Pakistan
KJ839893	Pakistan
KJ840382	Pakistan
KJ840367	Pakistan
KJ840065	Pakistan
KJ839964	Pakistan
KJ840058	Pakistan
KJ839945	Pakistan
KJ839888	Pakistan
KJ840353	Pakistan
KJ840283	Pakistan
KJ840096	Pakistan
KJ840032	Pakistan
KJ840041	Pakistan
KJ840015	Pakistan
KJ840309	Pakistan
KJ840395	Pakistan
KJ840489	Pakistan
KJ840126	Pakistan
KJ840477	Pakistan
KJ840075	Pakistan
KJ839879	Pakistan
KJ840150	Pakistan
KJ840108	Pakistan
KJ840200	Pakistan
KJ840193	Pakistan
KJ840279	Pakistan

KJ840399	Pakistan
KJ840536	Pakistan
KJ839836	Pakistan
KJ840320	Pakistan
KJ840389	Pakistan
KJ839962	Pakistan
KJ840545	Pakistan
KJ840373	Pakistan
KJ840190	Pakistan
KJ840143	Pakistan
KJ839996	Pakistan
KJ840063	Pakistan
KJ839897	Pakistan
KJ840156	Pakistan
KJ840274	Pakistan
KJ840262	Pakistan
KJ840441	Pakistan
KJ839837	Pakistan
KJ840543	Pakistan
KJ840184	Pakistan
KJ840130	Pakistan
KJ840429	Pakistan
KJ839961	Pakistan
KJ840180	Pakistan
KJ840125	Pakistan
KJ840365	Pakistan
KJ839956	Pakistan
KJ840268	Pakistan
KJ840515	Pakistan
KJ840480	Pakistan
KJ839886	Pakistan
KJ840100	Pakistan
KJ839994	Pakistan
KJ840391	Pakistan
KJ840427	Pakistan
KJ839841	Pakistan
KJ840326	Pakistan
KJ840159	Pakistan
KJ840546	Pakistan
KJ840215	Pakistan
KJ839850	Pakistan
KJ839894	Pakistan
KJ840495	Pakistan
KJ840113	Pakistan
KJ840149	Pakistan
KJ840363	Pakistan
KJ839951	Pakistan

KJ840194	Pakistan
KJ839851	Pakistan
KJ839995	Pakistan
KJ840323	Pakistan
KJ840351	Pakistan
KJ840503	Pakistan
KJ840534	Pakistan
KJ840210	Pakistan
KJ840034	Pakistan
KJ840470	Pakistan
KJ840475	Pakistan
KJ840101	Pakistan
KJ840148	Pakistan
KJ839885	Pakistan
KJ840314	Pakistan
KJ840354	Pakistan
KJ840517	Pakistan
KJ840023	Pakistan
KJ840045	Pakistan
KJ839963	Pakistan
KJ840116	Pakistan
KJ840392	Pakistan
KJ840490	Pakistan
KJ840201	Pakistan
KJ840174	Pakistan
KJ840229	Pakistan
KJ840456	Pakistan
KJ840203	Pakistan
KJ840446	Pakistan
KJ840486	Pakistan
KJ839984	Pakistan
KJ840525	Pakistan
KJ840153	Pakistan
KJ840050	Pakistan
KJ839862	Pakistan
KJ840224	Pakistan
KJ840518	Pakistan
KJ839843	Pakistan
KJ840205	Pakistan

Suppl. 1. COI and cytb based species delimitation of Pediculus humanus complex using online version of PTP (http://species.h-its.org/ptp/) (Job ID 7183)

I. COI-5′ (barcode) PTP analysis including *Pediculus schaeffi* as outgroup.

1. Maximum likelihood solution:

```
# Max likilhood partition
Species 1 (support = 1.000)
     'AY695999_P. schaeffi_1'

Species 2 (support = 1.000)
     'KC241887_P. schaeffi_2'

Species 3 (support = 0.532)

'AY239288_B','EU493425_B','EU493371_B','EU493369_B','EU493374_B','EU493362_B'
,'EU493436_B','KJ840492_B','EU493419_B','EU493444_B','AY695950_B','EU493375_B
','KJ840331_B','AY695945_B'

Species 4 (support = 0.921)

'KC685845_A','KJ840487_A','HM357241_A','KJ840004_A','KJ839972_A','KJ840310_A'
,'KJ840290_A','EU493383_A','AY589981_A','AY590021_A','AY695956_A','AY695955_A
','EU493392_A','AY695990_A','AY589984_A','AY239287_A','AY590023_A','AY695987_
A','AY590022_A','AY590004_A','AY589973_A','AY590017_A','AY239285_A'

Species 5 (support = 0.550)
     'KC685846_E','KC685842_E'

Species 6 (support = 1.000)
     'KC685849_C'

Species 7 (support = 0.992)

'KJ840051_D','KJ840421_D','KJ840070_D','KJ840414_D','KJ840490_D','KJ840185_D'
,'KJ840222_D','KJ840220_D','AY590041_D','KJ840456_D','KJ839866_D','KJ840075_D
','KJ840543_D','KJ840321_D','KJ840144_D','KJ840041_D'
```

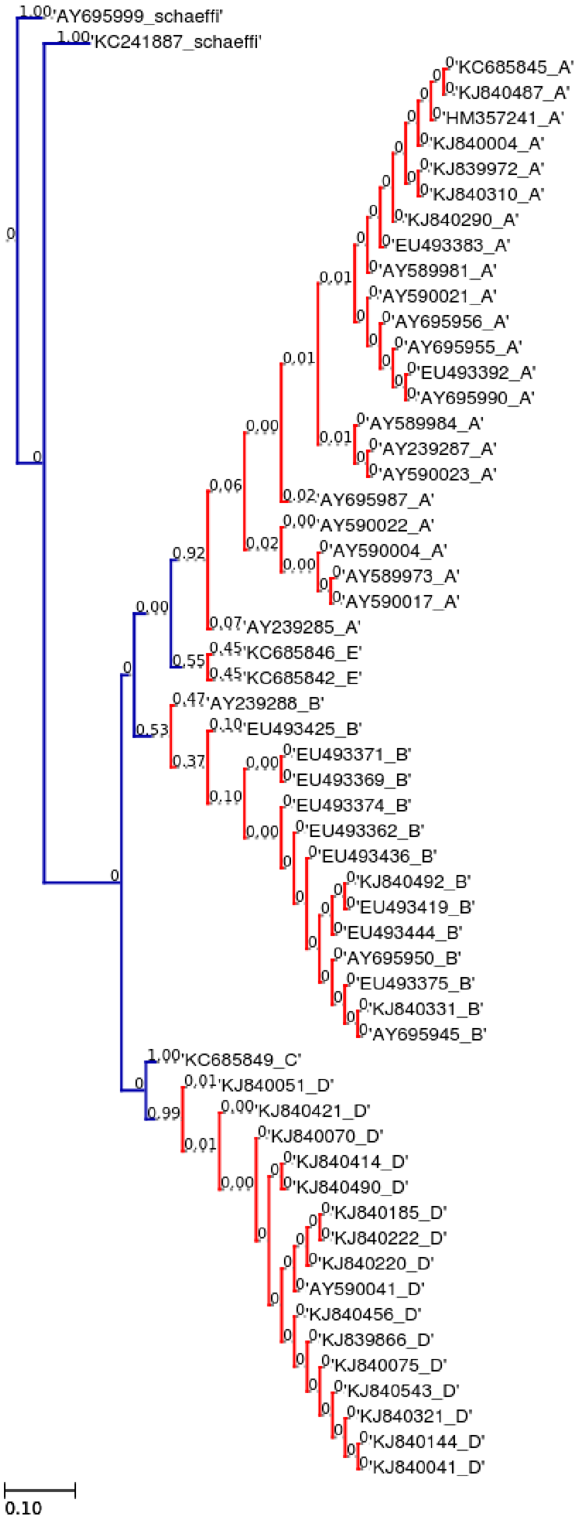
'AY695999_schaeffi'
'KC241887_schaeffi'
'KC685845_A'
'KJ840487_A'
'HM357241_A'
'KJ840004_A'
'KJ839972_A'
'KJ840310_A'
'KJ840290_A'
'EU493383_A'
'AY589981_A'
'AY590021_A'
'AY695956_A'
'AY695955_A'
'EU493392_A'
'AY695990_A'
'AY589984_A'
'AY239287_A'
'AY590023_A'
'AY695987_A'
'AY590022_A'
'AY590004_A'
'AY589973_A'
'AY590017_A'
'AY239285_A'
'KC685846_E'
'KC685842_E'
'AY239288_B'
'EU493425_B'
'EU493371_B'
'EU493369_B'
'EU493374_B'
'EU493362_B'
'EU493436_B'
'KJ840492_B'
'EU493419_B'
'EU493444_B'
'AY695950_B'
'EU493375_B'
'KJ840331_B'
'AY695945_B'
'KC685849_C'
'KJ840051_D'
'KJ840421_D'
'KJ840070_D'
'KJ840414_D'
'KJ840490_D'
'KJ840185_D'
'KJ840222_D'
'KJ840220_D'
'AY590041_D'
'KJ840456_D'
'KJ839866_D'
'KJ840075_D'
'KJ840543_D'
'KJ840321_D'
'KJ840144_D'
'KJ840041_D'
0.10

2. Highest Bayesian supported solution:

```
# Most supported partition found by simple heuristic search
Species 1 (support = 1.000)
     'AY695999_P. schaeffi_1'

Species 2 (support = 1.000)
     'KC241887_P. schaeffi_2'

Species 3 (support = 0.532)

'AY239288_B','EU493425_B','EU493371_B','EU493369_B','EU493374_B','EU493362_B'
,'EU493436_B','KJ840492_B','EU493419_B','EU493444_B','AY695950_B','EU493375_B
','KJ840331_B','AY695945_B'

Species 4 (support = 1.000)
     'KC685849_C'

Species 5 (support = 0.992)

'KJ840051_D','KJ840421_D','KJ840070_D','KJ840414_D','KJ840490_D','KJ840185_D'
,'KJ840222_D','KJ840220_D','AY590041_D','KJ840456_D','KJ839866_D','KJ840075_D
','KJ840543_D','KJ840321_D','KJ840144_D','KJ840041_D'

Species 6 (support = 0.921)

'KC685845_A','KJ840487_A','HM357241_A','KJ840004_A','KJ839972_A','KJ840310_A'
,'KJ840290_A','EU493383_A','AY589981_A','AY590021_A','AY695956_A','AY695955_A
','EU493392_A','AY695990_A','AY589984_A','AY239287_A','AY590023_A','AY695987_
A','AY590022_A','AY590004_A','AY589973_A','AY590017_A','AY239285_A'

Species 7 (support = 0.550)
     'KC685846_E','KC685842_E'
```

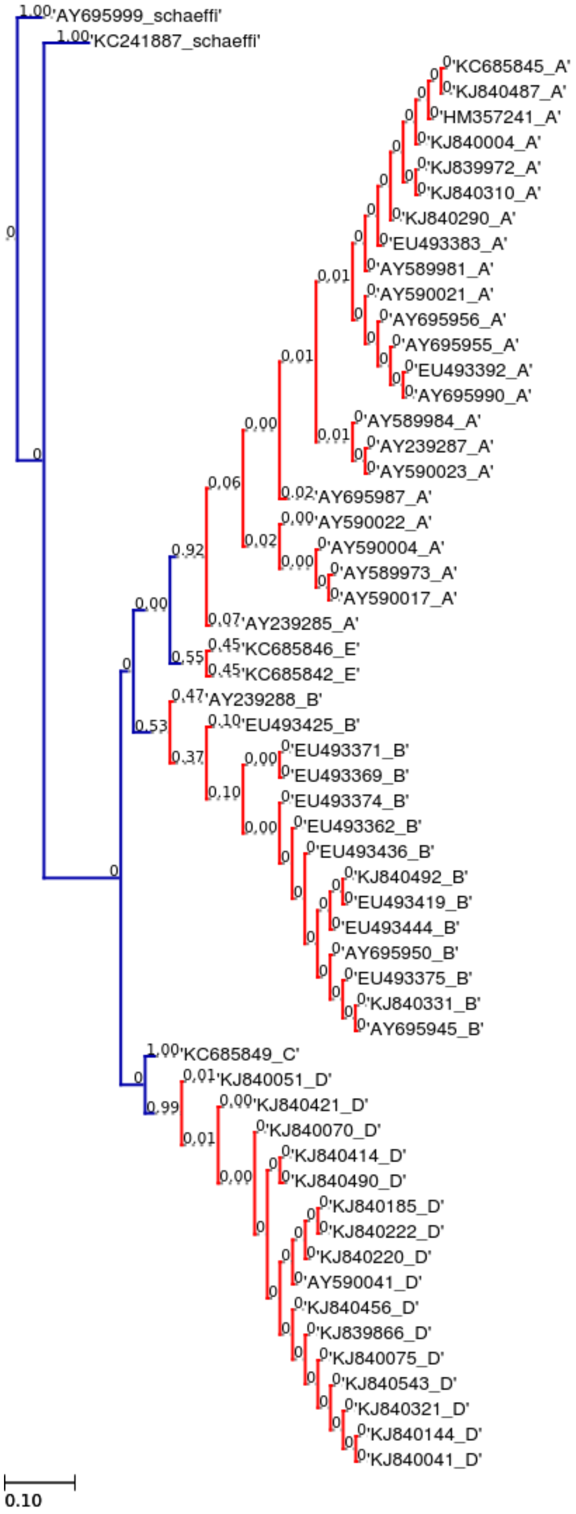
'AY695999_schaeffi'
'KC241887_schaeffi'
'KC685845_A'
'KJ840487_A'
'HM357241_A'
'KJ840004_A'
'KJ839972_A'
'KJ840310_A'
'KJ840290_A'
'EU493383_A'
'AY589981_A'
'AY590021_A'
'AY695956_A'
'AY695955_A'
'EU493392_A'
'AY695990_A'
'AY589984_A'
'AY239287_A'
'AY590023_A'
'AY695987_A'
'AY590022_A'
'AY590004_A'
'AY589973_A'
'AY590017_A'
'AY239285_A'
'KC685846_E'
'KC685842_E'
'AY239288_B'
'EU493425_B'
'EU493371_B'
'EU493369_B'
'EU493374_B'
'EU493362_B'
'EU493436_B'
'KJ840492_B'
'EU493419_B'
'EU493444_B'
'AY695950_B'
'EU493375_B'
'KJ840331_B'
'AY695945_B'
'KC685849_C'
'KJ840051_D'
'KJ840421_D'
'KJ840070_D'
'KJ840414_D'
'KJ840490_D'
'KJ840185_D'
'KJ840222_D'
'KJ840220_D'
'AY590041_D'
'KJ840456_D'
'KJ839866_D'
'KJ840075_D'
'KJ840543_D'
'KJ840321_D'
'KJ840144_D'
'KJ840041_D'
0.10

I. Cytb PTP analysis including *Pediculus schaeffi* as outgroup.

Maximum likelihood solution:

```
# Max likilhood partition
Species 1 (support = 1.000)
     'AY696067_P_schaeffi_1'

Species 2 (support = 1.000)
     'KC241883_P_schaeffi_2'

Species 3 (support = 0.991)
     'Hap_22_C'

Species 4 (support = 0.785)
     'Hap_10_D','Hap_9_D','Hap_6_D','Hap_7_D'

Species 5 (support = 0.811)
     'Hap_14_B','Hap_5_B','Hap_20_B','Hap_4_B','Hap_2_B','Hap_3_B'

Species 6 (support = 0.776)

'Hap_1_A','Hap_18_A','Hap_21_A','Hap_13_A','Hap_12_A','Hap_16_A','Hap_15_A','
Hap_8_A','Hap_17_A','Hap_19_A'

Species 7 (support = 0.825)
     'Hap_11_E','Hap_23_E'
```

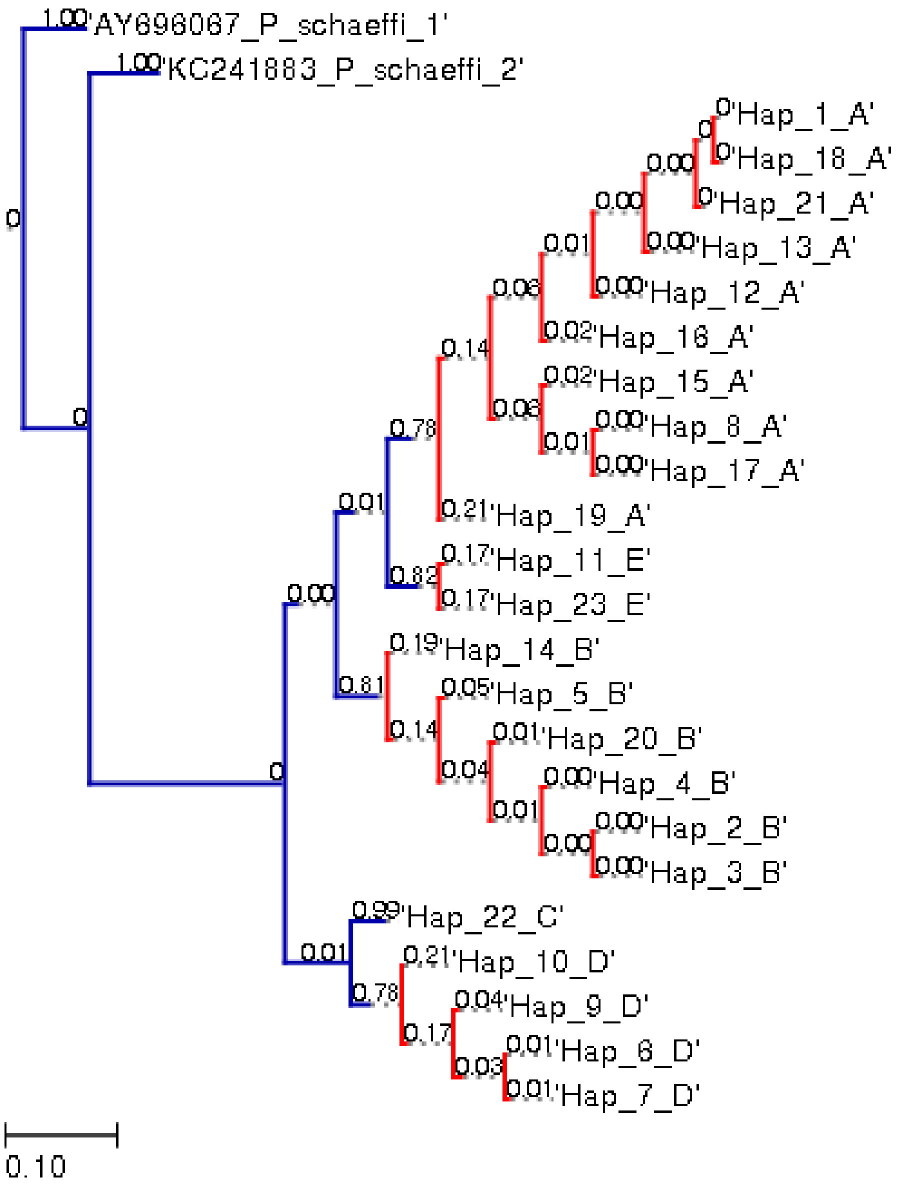

1.00 'AY696067_P_schaeffi_1'
1.00 'KC241883_P_schaeffi_2'
0 'Hap_1_A'
0 'Hap_18_A'
0 'Hap_21_A'
0.00 'Hap_13_A'
0.00 'Hap_12_A'
0.02 'Hap_16_A'
0.02 'Hap_15_A'
0.00 'Hap_8_A'
0.00 'Hap_17_A'
0.21 'Hap_19_A'
0.17 'Hap_11_E'
0.17 'Hap_23_E'
0.19 'Hap_14_B'
0.05 'Hap_5_B'
0.01 'Hap_20_B'
0.00 'Hap_4_B'
0.00 'Hap_2_B'
0.00 'Hap_3_B'
0.99 'Hap_22_C'
0.21 'Hap_10_D'
0.04 'Hap_9_D'
0.01 'Hap_6_D'
0.01 'Hap_7_D'
0.10

Highest Bayesian supported solution:

```
# Most supported partition found by simple heuristic search
Species 1 (support = 1.000)
     'AY696067_P_schaeffi_1'

Species 2 (support = 1.000)
     'KC241883_P_schaeffi_2'

Species 3 (support = 0.811)
     'Hap_14_B','Hap_5_B','Hap_20_B','Hap_4_B','Hap_2_B','Hap_3_B'

Species 4 (support = 0.991)
     'Hap_22_C'

Species 5 (support = 0.785)
     'Hap_10_D','Hap_9_D','Hap_6_D','Hap_7_D'

Species 6 (support = 0.776)

'Hap_1_A','Hap_18_A','Hap_21_A','Hap_13_A','Hap_12_A','Hap_16_A','Hap_15_A','
Hap_8_A','Hap_17_A','Hap_19_A'

Species 7 (support = 0.825)
     'Hap_11_E','Hap_23_E'
```

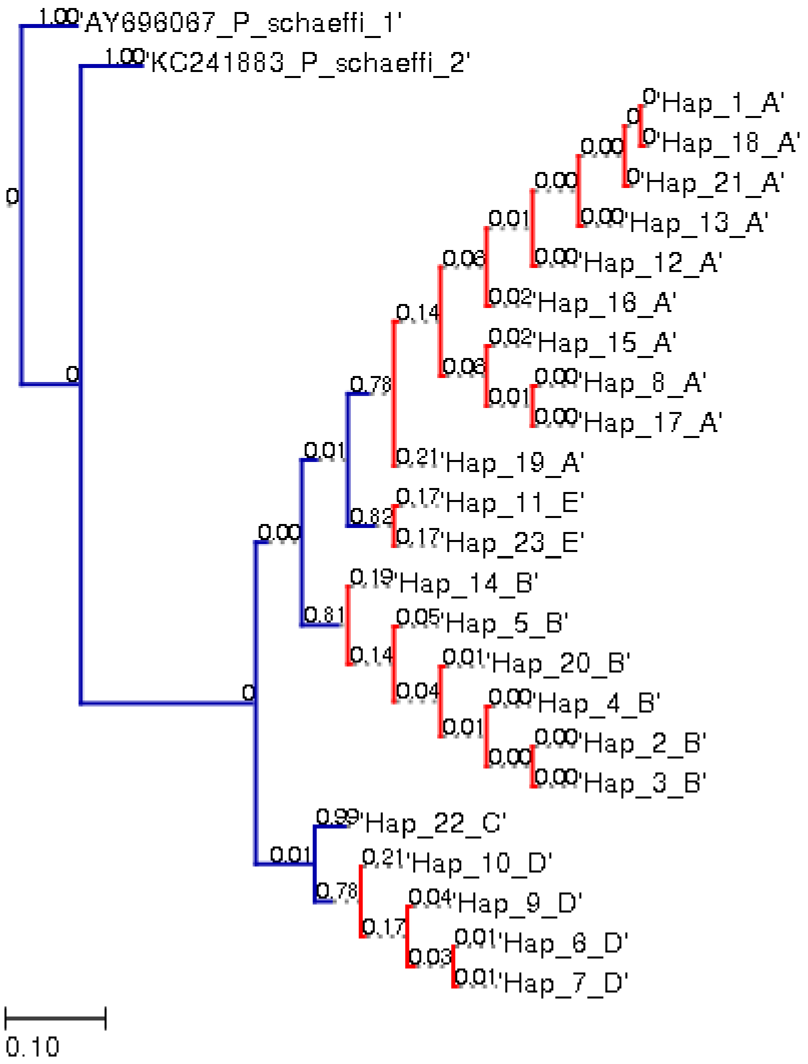
'AY696067_P_schaeffi_1'
'KC241883_P_schaeffi_2'
'Hap_1_A'
'Hap_18_A'
'Hap_21_A'
'Hap_13_A'
'Hap_12_A'
'Hap_16_A'
'Hap_15_A'
'Hap_8_A'
'Hap_17_A'
'Hap_19_A'
'Hap_11_E'
'Hap_23_E'
'Hap_14_B'
'Hap_5_B'
'Hap_20_B'
'Hap_4_B'
'Hap_2_B'
'Hap_3_B'
'Hap_22_C'
'Hap_10_D'
'Hap_9_D'
'Hap_6_D'
'Hap_7_D'
0.10
